# Expression of circulating miRNAs associated with lymphocyte differentiation and activation in CLL—another piece in the puzzle

**DOI:** 10.1007/s00277-016-2840-6

**Published:** 2016-10-12

**Authors:** Agata A. Filip, Anna Grenda, Sylwia Popek, Dorota Koczkodaj, Małgorzata Michalak-Wojnowska, Michał Budzyński, Ewa Wąsik-Szczepanek, Szymon Zmorzyński, Agnieszka Karczmarczyk, Krzysztof Giannopoulos

**Affiliations:** 1 Department of Cancer Genetics, Medical University of Lublin, Radziwiłłowska 11, 20-080 Lublin, Poland; 2 Department of Hematooncology and Bone Marrow Transplantation, Medical University of Lublin, Lublin, Poland; 3 Department of Experimental Hematooncology, Medical University of Lublin, Lublin, Poland

**Keywords:** CLL, Circulating miRNA, Microenvironment, Prognostic factors, Lymphocyte differentiation

## Abstract

Expression of microRNAs is altered in cancer. Circulating miRNA level assessed in body fluids commonly reflects their expression in tumor cells. In leukemias, however, both leukemic and nonleukemic cells compose circulating miRNA expression profile of peripheral blood. The latter contribution to extracellular miRNA pool may result in specific microenvironmental signaling, which promotes proliferation and survival. In our study, we used qT-PCR to assay peripheral blood serum of 22 chronic lymphocytic leukemia (CLL) patients for the expression of 84 miRNAs associated with activation and differentiation of B and T lymphocytes. Results were analyzed regarding the most important prognostic factors. We have found that the general expression of examined miRNAs in CLL patients was lower as compared to healthy volunteers. Only miR-34a-5p, miR31-5p, miR-155-5p, miR-150-5p, miR-15a-3p, and miR-29a-3p were expressed on a higher level. Alterations of expression observed in CLL patients involved miRNAs associated both with B and T lymphocyte differentiation and activation. The most important discriminating factors for all functional miRNA groups were trisomy 12, CD38 expression, B2M level, WBC, and *NOTCH1* gene mutation. Correlation of expression of miRNAs related to T lymphocytes with prognostic factors proves their supportive function in a leukemic microenvironment. Further studies utilizing a larger test group of patients may warrant the identification of circulating miRNAs that are key players in intercellular interactions and should be considered in the design of microenvironment-targeted therapies.

**Electronic supplementary material:**

The online version of this article (doi:10.1007/s00277-016-2840-6) contains supplementary material, which is available to authorized users.

## Introduction

Chronic lymphocytic leukemia (CLL) is one of the most common leukemias of the elderly. It is characterized by the accumulation of mature leukemic B lymphocytes in peripheral blood, bone marrow, and lymphatic organs. Leukemic cells are resistant to apoptosis, both spontaneous and induced with antileukemic drugs. This phenomenon is associated with the increased expression of antiapoptotic genes like *BCL2* or *MCL1* and decreased proapoptotic proteins (i.e., TP53) involvement [1]. The above abnormalities may result from gene mutations and improper gene expression caused by posttranscriptional alterations, or may be an indirect consequence of prosurvival signaling from the microenvironment composed of mesenchymal stromal cells, nurse-like cells (NLCs), T lymphocytes, and others.

Clonal expansion of CLL lymphocytes begins with B cell precursor stimulation by the antigen within or outside the germinal centers, while the driver antigens targeting B cell receptor are thought to be foreign or—more probably—autologous [2]. Clinically, CLL progresses with profound immunodeficiency, being the common cause of disease complications and death. The above facts point to the unique nature of CLL on the crossroads of oncogenesis and autoimmunity [3]. T lymphocyte population is involved as well; CLL patients display T cell dysfunctions and the observed higher than normal absolute regulatory T cells (Tregs) number is considered responsible for suppressing the antitumor immune response [4].

MicroRNAs (miRNAs) regulate gene expression at the posttranscriptional level that make them the key players in all biological processes [1]. By regulation of oncogenes and tumor suppressors, miRNAs contribute to oncogenesis, and it was proven that the miRNA expression pattern better characterized cancer cells than messenger RNA (mRNA) expression, so potentially they may serve as diagnostic, prognostic, and predictive factors [5–7]. What is more, intact miRNA molecules freely circulate in body fluids like blood, milk, saliva, or urine [8]. In many pathologic conditions, the circulating miRNA level reflects the level of miRNA expression in affected tissue. Once they were thought to be incidentally released from the cells (i.e., from the cells dying by necrosis), now the new function of circulating miRNAs was discovered: they are actively secreted molecules shuttling between cells as mediators [9]. Indeed, miRNAs may be transferred from cell to cell packaged into exosomes, shedding vesicles or apoptotic bodies. Another way of extracellular horizontal miRNA transfer is their binding to lipoproteins (i.e., high-density lipoprotein, HDL) or to proteins like AGO2 and NPM1 [10]. This way, the small molecules may affect gene expression in target cells.

Studies in CLL microenvironment have shown that the relation between leukemic and bystander cells is mutual, for NLCs differentiate in vitro exclusively when cultured with leukemic lymphocytes, not normal CD19+ B lymphocytes [11]. To date, only indirect cell-cell contact and chemokines produced by microenvironmental cells were considered responsible for their interaction and observed protective properties toward CLL lymphocytes. However, it is more than likely that specific miRNAs transferred from bystander cells may also matter.

CLL may manifest as indolent, chronic disease or as aggressive disorder associated with markedly shortened lifespan [8]. Each type requires different treatment strategies. As the Rai and Binet clinical staging systems turned out insufficient for the precise stratification of patients to low- and high-risk groups, intensive research in the field led to the identification of several relevant molecular prognostic and predictive factors [12, 13]. First gross discoveries included selected recurrent chromosomal aberrations, i.e., trisomy 12, del 13q14, del 11q22.3-q23.1, and del 17p [14]. All of these altered regions contain important genes whose contribution to CLL is more or less confirmed to date [15–17]. The most common 13q14 deletion as the monoallelic sole abnormality confers favorable prognosis, although it covers *loci* for two miRNAs, miR-15a and miR-16-1, which target the *BCL2* gene [17]. The presence of del 11q22.3-q23.1 (*ATM locus*) or del 17p (*TP53 locus*) defines high-risk group patients characterized by decreased overall survival (OS) and resistance to chemotherapy based on alkylating agents [14, 18]. Since CLL lymphocytes manifest low mitotic activity in vitro, chromosomal analyses were not very successful until the introduction of fluorescent in situ hybridization (FISH) into routine diagnostics. FISH significantly increased sensitivity of cytogenetics, making it possible to identify chromosomal abnormalities in up to 80 % of CLL patients [19].

Next, CLL prognostic milestones were mutations of the genes encoding the variable part of the immunoglobulin heavy chain (IgVH) [20]. Patients with unmutated *IgVH* genes were shown to poorly respond to multiregimen chemotherapy and had significantly shorter OS [20]. As *IgVH* gene sequencing is complicated and time-consuming, more available surrogate markers were identified. CD38 expression on ≥30 % CLL lymphocytes or ZAP70 expression found in ≥20 % of leukemic cells is recognized as poor prognostic markers, partially reflecting unmutated IgVH status [20–22].

Recent whole genome and whole exome sequencing experiments brought other pieces of the puzzle for our understanding of CLL development. Many cryptic recurrent mutations were found, involving *TP53*, *NOTCH1*, *SF3B1*, *BIRC3*, *XPO1*, *MYD88*, *KLHL6* gene, and others [23–26]. The biological role of selected cryptic lesions in leukemogenesis was reviewed before; they clearly influence the course of the disease as well [27]. Mutations of *TP53* gene commonly coexist with 17p deletions; however, their sole presence (4.5 % CLL patients) typifies poor survival similar to sole 17p deletions and bi-allelic *TP53* alterations [18]. This discovery enables more precise stratification of patients, as well as the knowledge concerning poor prognostic impact of *NOTCH1* mutation, typically coexisting with trisomy 12, the chromosomal aberration which otherwise confers intermediate prognosis for patients with mutated *IgVH* genes [28].

Some studies formulate new CLL classifications based on genetic findings. Rossi and colleagues recognize four prognostic groups (high, intermediate, low, and very low risk) with 10-year survival 29, 37, 57, and 69.3 %, respectively [26]. They integrated cytogenetic and mutational analysis considering del 13q14, deletion or mutation of *TP53*, trisomy 12, del 11q22-q23 together with *NOTCH1*, *BIRC3*, and *SF3B1* mutations [26]. The status of *IGVH* and *TP53* genes is considered crucial in recent five-variable International Prognostic Index for Patients with CLL (CLL-IPI) proposed by The International CLL-IPI working group [29]. There are also some trials of miRNA profiling in CLL patients to select these of prognostic significance [30]. The elegant study by Visone and coworkers presents the results of miRNA expression assessment in the context of chromosomal abnormalities [31]. All factors considered to date, however, are intrinsic factors, inseparably associated with leukemic lymphocytes.

Despite the progress in the design of treatment strategies, the aggressive form of CLL is still incurable; thus, the research in the field of microenvironmental factors concurring in leukemogenesis is important both for diagnostic and therapeutic purposes [1].

The aim of the presented study was the assessment of expression of circulating miRNAs most relevant to specific stages of T cell and B cell development in CLL patients. The results were then related to clinical and biochemical patients’ characteristics as well as to cytogenetic and molecular prognostic factors.

## Materials and methods

### Patients

With informed consent, in accordance with the Declaration of Helsinki and approval from the Medical University of Lublin Bioethics Committee (KE-0254/155/2010 and KE-0254/118/2011), peripheral blood was obtained from 36 consecutive, treatment naive patients, diagnosed with CLL at the Department of Hematooncology and Bone Marrow Transplantation, Medical University of Lublin. After serum miRNA isolation, 22 patients (10 women and 12 men) were qualified for further analyses.

By reference to the Rai classification, seven patients were at stage 0, seven at stage I, six at stage II, one at stage III, and one patient at stage IV. Basic clinical and hematological parameters are shown in Table 1, and detailed patients’ characteristics are reported in Supplementary Table S1. Peripheral blood from eight healthy volunteers (three women and five men, aged 22–45) attending the Regional Blood Donation and Hemotherapy Center in Lublin served as the reference sample.

**Table 1:** Characteristics of CLL patients

	*N* = 22 (100 %)
Sex	
Male	12 (54 %)
Female	10 (46 %)
Age (years)	
Mean	65.2
Median	64
Range	50–85
Rai stage	
0	7 (32 %)
I	7 (32 %)
II	6 (27 %)
III	1 (4.5 %)
IV	1 (4.5 %)
WBC (×10^9^/L)	
Mean	73.10
Median	35.72
Range	14.29–742
ALC (×10^9^/L)	
Mean	64.63
Median	28.52
Range	7.06–721
PLT (×10^9^/L)	
Mean	206.54
Median	199.0
Range	124–373
B2M (mg/L)	
Mean	4.04
Median	3.08
Range	1.39–10.5
LDH (IU/L)	
Mean	467.67
Median	404.5
Range	316–985
CD38+	7/22 (31.8 %)
ZAP70+	8/22 (36 %)
*IGVH-*mut	6/21 (28.6)
*NOTCH1-*mut	5/22 (22.7 %)
*TP53-*mut	5/21 (23.8 %)
FISH	
Normal	2/22 (9 %)
del13q14/*RB1*	4/22 (18 %)
del13q14.3/D13S319	15/22 (68 %)
del17p/*TP53*	8/22 (36 %)
del11q/*ATM*	8/22 (36 %)
+12	6/22 (27 %)

*WBC* white blood cells count, *ALC* absolute lymphocyte count, *PLT* absolute platelet count, *B2M* beta-2-microglobulin, *LDH* lactate dehydrogenase, *mut* mutation

### Immunophenotyping

Whole blood specimens collected in EDTA (Sarstedt tubes, Sarstedt, Germany) were immunophenotyped by flow cytometry with FACSCalibur apparatus equipped with the CellQuest software (Becton-Dickinson Immunocytometry System); 10,000 cells for each sample were assessed after incubation with monoclonal mouse antihuman CD5-PE, CD19-PE-Cy5, CD23-FITC, CD38-FITC, and ZAP70 antibodies along with appropriate isotype controls (all from BD Bioscience). Antibodies were applied at 1 μg/100 μL of cell suspension (1 × 10^6^ cells in 1 % BSA/PBS), and samples were processed according to the manufacturer’s instructions.

### Cell cultures and FISH analysis

Peripheral blood samples were collected in Monovette heparin tubes (Sarstedt, Germany). Mononuclear cells were isolated by gradient density centrifugation (Lymphoprep™, AXIS-SHIELD). Stimulated cell cultures were prepared using RPMI 1640 medium with l-glutamine supplemented with 20 % FCS vol/vol (Biomed Poland), 10 U/mL penicillin, and 50 μg/mL streptomycin (Gibco, Invitrogen). The cultures were stimulated with pokeweed mitogen, 2.5 μg/mL (PWM, Sigma), or TPA, 10 ng/mL (12-O-tetradecanoylphorbol 13-acetate, Sigma-Aldrich). Cells were cultured at 37 °C in a humidified incubator in 5 % CO_2_ atmosphere for 72 h and 5 days. The cultures were harvested conventionally, and the metaphase spreads were stained using GTG and RHG banding techniques.

FISH was performed utilizing the following probes: Vysis D13S319 (13q14.3), Vysis LSI 13 (RB1), Vysis LSI ATM, CEP12, and Vysis LSI TP53 SpectrumOrange/CEP 17 SpectrumGreen Probe (all from Abbott Molecular, USA), strictly according to the manufacturer’s instructions; 200 interphase cells were examined for each patient. The established cutoff levels were as follows: 5 % positive cells for trisomy 12, 8 % for del(13)(q14.3) and *RB1 locus* deletion, and 9 % for both del(11)(q22.3) and del(17)(p13.1).

### DNA isolation and sequencing

Genomic DNA was extracted from 200 μL peripheral blood samples collected in EDTA with GeneMATRIX Quick Blood DNA Purification Kit (EURx, Poland), according to the manufacturer’s manual. DNA quality and quantity were assessed by means of NanoDrop 2000 spectrophotometer (ThermoScientific) and by agarose gel electrophoresis (2 % agarose in TBE). For further investigation, samples containing nondegraded, high molecular weight DNA at the concentration of at least 100 ng/μL, with A260/A280 ratio between 1.8 and 2.0, were used.

The most common *NOTCH1* mutations located in fragment of exon 34 (c.7544-7545delCT, c.7225C>T, c.7369C>G, c.7446delC, c.7392delC, c.7321C>T, c.7507C>T, c.7244-7271del28) were analyzed by direct sequencing. DNA was amplified with high fidelity Advantage HD polymerase (Clontech). All primers and conditions were designed at the Department of Cancer Genetics, Medical University of Lublin (sequences and detailed conditions are available on request).

*TP53* mutational status was determined by automated sequencing of exons 4–10, strictly according to the protocol published by the International Agency for Research on Cancer (IARC) [32, 33]. For PCR reactions, 50 ng DNA and Color Perpetual OptiTaq PCR Master Mix (EURx, Gdansk, Poland) were used.

The immunoglobulin heavy chain variable (IGHV) mutation status was determined by DNA amplification with polymerase chain reaction (PCR) using six different sense primers specific for framework region (FR) 1 consensus family (IGHV1–IGHV6) and one antisense primer complementary to the germline JH regions, according to the BIOMED-2 Concerted Action protocols [34]. The seven IGVH families were amplified in six individual PCRs. PCR products were separated on 2 % agarose gel, cut out, and purified using QIAquick Gel Extraction Kit (Qiagen, Hilden, Germany). Purified PCR products were directly sequenced using an ABI PRISM BigDye Terminator v3.1 cycle sequencing kit (Applied Biosystems, Foster City, CA, USA), following the manufacturer’s instructions by means of an automatic ABI 3500 Genetic Analyzer (Applied Biosystems, Foster City, CA, USA). The percentage of hypermutations was calculated. Each clonal DNA *IGHV* sequence was aligned with the closest germline sequence using the international ImMunoGeneTics information system (IMGT, http://www.imgt.org/). The sequences with a germline homology 98 % or higher were considered unmutated, and those with a homology less than 98 % as mutated.

### MicroRNA isolation and profiling

Peripheral blood samples were collected in S-Monovette Serum Gel Z tubes (Sarstedt, Germany) and immediately placed at 4 °C for 30–40 min, in order to facilitate coagulation. Samples were then centrifuged at 2000 rpm for 20 min at RT to spin down the clot. The separated supernatant was carefully transferred into fresh Eppendorf tubes and centrifuged again at 4000 rpm for 10 min at 4 °C. Carefully collected transparent serum samples were then aliquoted into fresh tubes and stored at −80 °C.

Total RNA including miRNA and small RNA fraction was isolated using miRNeasy Serum/Plasma Kit (Qiagen) according to the manufacturer’s instructions. Serum/Plasma Spike-In Control (Qiagen) was added to provide system for normalization. Quality and quantity of RNA were assessed by means of NanoDrop 2000. Samples containing at least 5 ng/μL of RNA, with A260/A280 ratio between 1.7 and 2.1, were used for further analysis. Isolated RNA was stored at −80 °C.

MicroRNA expression profiling was performed by means of T-Cell & B-Cell Activation miScript miRNA PCR Array (Qiagen, cat. # MIHS-111Z), using primers for 84 preselected miRNAs, important for differentiation, maturation, and activation of B and T lymphocytes (see Supplementary Table S3 for the full list).

Complementary DNA (cDNA) synthesis was carried out utilizing miScript II RT Kit (Qiagen) on 125 ng of RNA in 20 μL of final volume. HiSpec buffer (Qiagen) was used for the selective conversion of mature miRNAs. After reverse transcription, reaction samples were stored at −20 °C. For qT-PCR, the miScript® SYBR® Green PCR Kit together with T-Cell & B-Cell Activation miScript miRNA PCR Array (both from Qiagen) were used according to the manufacturer’s instructions. Each RT sample was diluted with 200 μL RNase free water, and 100 μL of the obtained cDNA solution was used as template for qT-PCR performed on a 96-well plate. The following snoRNAs—SNORD95, SNORD96A, and RNU6B/RNU6-2—were used as a normalization control for the array data. Total qT-PCR reaction volume was 25 μL/well. qPCR was performed by means of 7500 Fast Real-Time PCR System (Applied Biosystems, USA). All reactions were run in duplicate.

Amplification curves were analyzed to define Ct (threshold cycle). The melting curve analysis was implemented. The relative expression of serum miRNA in CLL patients was assessed with the 2^−ΔΔCt^ method, using pooled miRNA from healthy donors as the reference [35].

### Statistical analyses

Statistical analyses were performed with Statistica 10.0 PL software and a free accessible R statistical package (www.R-project.org).

For array data analysis, the original data were normalized and expressed in logarithmic scale. For comparison of pairs of groups, Welch’s corrected *t* test was applied. The significance of diversification in the two groups was assessed by a variance analysis test (test *F*).

For the other analyses, the normality of data distribution was tested by means of the Shapiro-Wilk test. Descriptive statistical analysis was performed utilizing median, minimal, and maximal values. The significance of differences between dependent samples was tested by means of the Wilcoxon matched pairs test and Kruskal-Wallis test and differences between independent samples were tested by the Mann-Whitney *U* test. The strength of interdependency of two variables was expressed with Spearman’s rank correlation coefficient (*R*).

The differences were considered statistically significant with *P* values less than 0.05.

## Results

### Clinico-molecular characteristics and cytogenetic findings

The final study group included 22 CLL patients. Their age ranged 50–85 (median 64), WBC ranged 14.29–742 × 10^9^/L (median 35.72×10^9^/L), absolute lymphocyte count ranged 7.06–721 × 10^9^/L (median 28.52×10^9^/L), and platelet count ranged 124–373 × 10^9^/L (median 199.0×10^9^/L) (Table 1, Online Resource Table S1). Seven patients were considered as CD38+ (the cutoff 30 % of positive cells) and eight patients as ZAP70+ (the cutoff 20 % of positive cells). In compliance with the Rai stage of the disease, 7 patients were in the favorable prognostic group, 13 patients in the intermediate, and 2 in the adverse prognostic group. The time of follow-up was 30 months; in 10/22 patients (45.5 %), treatment has to be initiated and time to treatment (TTT) ranged from 0 to 27 months. Three patients died of CLL during follow-up; 2.5-year survival was 80.36 %.

Successful *IGVH* gene sequencing was performed in 21 patients of whom 6 (28.6 %) were identified with mutation (the concordance with germinal sequence ranged 89.45–95.77 %).

**Fig. 1:**
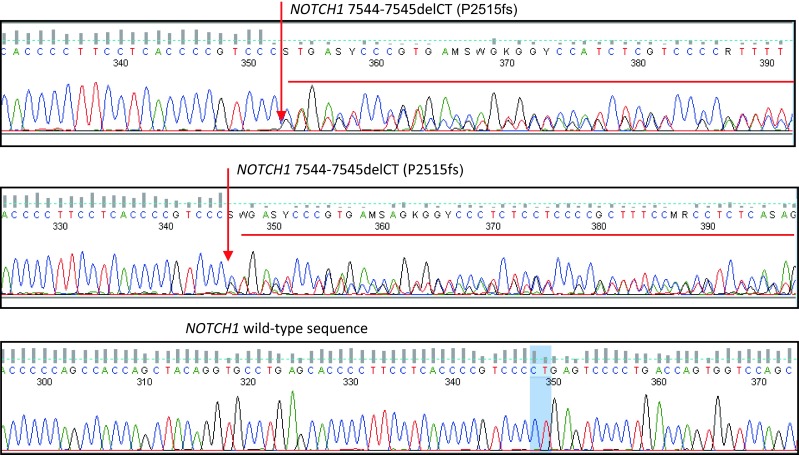
Exemplary sequencing results of *NOTCH1* gene in CLL patients. *Arrows* mark the site of mutation within the *NOTCH1* gene (exon 34)

*NOTCH1* mutation c.7544-7545delCT (P2515fs), located in exon 34, was found in five patients (22.7 %) (Fig. 1). Other *NOTCH1* mutations within this region were not identified. *IGVH* and *NOTCH1* mutations were mutually exclusive (Online Resource Table S1).

*TP53* mutations were found in 5 cases out of 21, including four substitutions (c.518T>A, c.673T>C, c.919C>G, c.673-36G>C) and a deletion c.778del26, as compared to reference sequence NM_000546.5.

FISH was aimed at the identification of four typical CLL aberrations (del11q, del13q14, del17p, and trisomy 12). As the prognosis of CLL patients was found to differ depending on the type of chromosome 13q deletion, we have decided to check both for deletion at D13S319 (13q14.3) and at *RB1 locus* [36].

FISH analysis revealed normal karyotype in two patients (Table 1, Table S1). Single aberration was identified in 7 patients (32 %), two coexisting aberrations were found in 9 patients (41 %), three in 3 patients, and 2 patients carried four aberrations simultaneously. We have found the deletion of D13S319 region (13q14.3) in 15 patients (68 %), while the deletion of *RB1* gene *locus* (13q14) was identified in 4 patients. The deletion of *TP53* gene *locus* (17p13) and the deletion of *ATM* gene *locus* (11q22-q23) were present in eight patients each. Trisomy of chromosome 12 was found in six patients, and in three of them, *NOTCH1* mutation was identified (Table 1, Online Resource Table S1).

Two patients with *TP53* deletion identified by FISH were found to carry mutation of the other allele of the gene. Altogether, 11/22 patients were characterized by mutation and/or deletion of the *TP53* gene (Table S1).

### MiRNA expression profiling

#### General serum miRNA expression of CLL patients is lower than their normal counterparts

Serum expression of the analyzed circulating miRNA in CLL patients was found to be much lower as compared with healthy individuals (Online Resource Table S2). Considering mean expression values, only 6 out of 84 analyzed miRNAs (7.14 %) were expressed at higher than normal level, while expression of two (2.38 %) was similar to normal. Among the miRNAs of the highest expression were miR-34a-5p (mean 6.6476 compared to 1 in normal individuals, median 1.8623), miR-31-5p (mean 4.4744, median 1.9298), miR-155-5p (mean 3.6579, median 1.5426), miR-150-5p (mean 1.5197, median 0.8621), miR-15a-3p (mean 1.4331, median 0.6042), and miR-29a-3p (mean 1.2440, median 0.9617) (Table 2).

**Table 2:** Circulating miRNAs of highest expression in CLL patients (fold change compared to the reference sample)

miRNA	Number	Mean	Median	Minimum	Maximum	Std. deviation
miR-34a-5p	22	6.647689	1.862355	0.000000	61.91293	14.16198
miR-31-5p	22	4.474437	1.929808	0.000000	26.27631	7.50078
miR-155-5p	22	3.657947	1.542619	0.000000	21.42856	4.858174
miR-150-5p	22	1.519764	0.862124	0.006600	8.65041	1.952950
miR-15a-3p	22	1.433167	0.604238	0.000000	12.88144	2.701575
miR-29a-3p	22	1.244012	0.961733	0.045252	5.97998	1.32323
miR-574-3p	22	0.968155	0.299874	0.028972	6.78702	1.58355
miR-29c-3p	22	0.929145	0.645566	0.009438	4.14237	0.97865

Expression values were presented as the logarithm of *R* to the base 2, where *R* was calculated as follows: R = 2^−ΔΔCt^, ΔΔCt = ΔCt of CLL patient − ΔCt of normal individuals, every ΔCt = Ct of miRNA examined − Ct of endogenous control, 1 − mean expression in pooled miRNAs of normal individuals

Serum miRNAs of the lowest expression included miR-147a (mean 0.0156, median 0.0025), miR-184 (mean 0.0328, median 0.0000), and miR-326 (mean 0.0690, median 0.0219) (Table 3).

**Table 3:** Circulating miRNAs of lowest expression in CLL patients (fold change compared to the reference sample)

miRNA	Number	Mean	Median	Minimum	Maximum	Std. deviation
miR-147a	22	0.015611	0.002544	0.000000	0.21399	0.046501
miR-184	22	0.032830	0.000000	0.000000	0.62236	0.132337
miR-326	22	0.060900	0.021956	0.000000	0.83752	0.17533
miR-18a-5p	22	0.103611	0.056592	0.000000	0.41750	0.113735
miR-195-5p	22	0.107020	0.057505	0.000458	0.54184	0.127800
miR-25-3p	22	0.109736	0.062301	0.001780	0.52281	0.13023
let-7d-5p	22	0.112145	0.058179	0.006802	0.442157	0.119731
miR-130b-3p	22	0.113208	0.062630	0.000000	0.461458	0.121767
miR-191-5p	22	0.116295	0.065128	0.004588	0.60537	0.150365
miR-30c-5p	22	0.127434	0.052519	0.008106	0.44780	0.14150
let-7e-5p	22	0.131160	0.063212	0.012399	0.513795	0.138210
miR-19a-3p	22	0.132547	0.087024	0.005614	0.48952	0.135557
miR-17-5p	22	0.132640	0.087608	0.005313	0.48162	0.128858
miR-16-5p	22	0.132781	0.079348	0.000449	0.77485	0.175928
miR-30b-5p	22	0.136220	0.076458	0.006022	0.42131	0.13321
miR-30d-5p	22	0.136391	0.075910	0.003105	0.56710	0.15640
miR23b-3p	22	0.137499	0.064416	0.003837	0.62679	0.16386
let-7f-5p	22	0.146194	0.072787	0.011085	0.552243	0.160592
let-7a-5p	22	0.147019	0.088026	0.005797	0.510367	0.155073
miR-182-5p	22	0.153754	0.052493	0.000000	1.10934	0.254892
miR-26a-5p	22	0.161983	0.087658	0.001916	0.67649	0.17815
miR-98-5p	22	0.173266	0.039254	0.000000	1.06964	0.29938
miR-142-5p	22	0.174660	0.086560	0.006512	0.61231	0.190252
miR-26b-5p	22	0.181229	0.098017	0.005669	0.71576	0.19998
miR222-3p	22	0.189829	0.091111	0.000000	1.13503	0.26830
miR-181c-5p	22	0.196126	0.099759	0.000000	0.94699	0.244802
miR-15b-5p	22	0.199360	0.078987	0.008995	1.47413	0.343739
miR-181b-5p	22	0.207683	0.088729	0.000000	0.90124	0.264372
miR-214-3p	22	0.212431	0.066792	0.000000	1.65352	0.36579
miR-24-3p	22	0.224084	0.095890	0.000000	1.39993	0.33267
miR-132-3p	22	0.237720	0.090168	0.000000	1.859543	0.402654
miR-27b-3p	22	0.244229	0.096800	0.027010	1.32214	0.36012
miR-17-3p	22	0.300520	0.084771	0.000000	1.40011	0.401672
miR-92a-3p	22	0.390319	0.049988	0.000600	5.51859	1.20007
miR-93-5p	22	0.439960	0.071626	0.000000	7.10019	1.49466
miR223-3p	22	0.446638	0.073717	0.000000	5.64799	1.18569

Expression values were presented as the logarithm of *R* to the base 2, where *R* was calculated as follows: R = 2^−ΔΔCt^, ΔΔCt = ΔCt of CLL patient − ΔCt of normal individuals, every ΔCt = Ct of miRNA examined − Ct of endogenous control, 1 − mean expression in pooled miRNAs of normal individuals

#### Prognostic factors mostly discriminating the general miRNA expression include the expression of CD38, status of chromosome 12 and RB1 locus, as well as patients’ age and B2M level

We analyzed circulating miRNA expression with regard to clinico-molecular and cytogenetic findings. There were no statistically significant differences between male and female patients. When we divided patients depending on age (up to 65 years and over), nine miRNAs were found expressed differently. The expression of miR-16-5p, miR-17-5p, miR-195-5p, miR-20a-5p, and miR-25-3p (in descending order) was significantly higher in younger patients, while miR-17-3p, miR-181c-5p, miR-181d-5p, and miR-182-5p expression was higher in older patients (Table 4). Three miRNAs—miR-125b-5p, miR-145-5p, and miR-99a-5p—were expressed at a lower level in patients with advanced disease (Rai stadium 2–4) when compared to patients diagnosed with Rai 0 and 1. We also observed the difference in three miRNA expression between patients depending on WBC: miR-125-5b, miR-128-3p, and miR-99a-5p were expressed lower in patients with WBC over 35 × 10^9^/L. In patients with high beta-2-microglobulin (B2M) level (3.0 mg/L and over), nine miRNAs were expressed lower than in the group of normal B2M: miR-7c, miR-125b-5p, miR-132-3p, miR-146b-5p, miR-18a-5p, miR-28b-3p, miR-365a-3p, miR-98-5p, and miR-99a-5p (Table 4). Only one miRNA was expressed differently between patients with LDH activity below or over 480 IU/L—lower miR-423-5p expression was observed in the latter group.

**Table 4:** Significant differences in circulating miRNA expression depending on prognostic markers and the need of treatment initiation

Expression	Age ≥65 years	Rai stages 2–4	WBC ≥35 × 10^9^/L	B2M ≥3 mg/L	CD38 ≥30 %	*NOTCH1* mutated	del *ATM* +	*TP53* del/mut +	del *RB1* +	Trisomy 12 −	Indications for treatment
↓	miR-16-5p miR-17-5p miR-195-5p miR-20a-5p miR-25-3p	miR-125b- 5p miR-145-5p miR-99a-5p	miR-125b- 5p miR-128-3p miR-99a-5p	miR-7c miR-125b-5p miR-132-3p miR-146b-5p miR-18a-5p miR-28b-3p miR-365a-3p miR-98-5p miR-99a-5p	let-7a-5p miR-7c let-7d-5p let-7e-5p let-7f-5p miR-101-3p miR-130b-3p miR-146b-5p miR-15a-5p miR-182-5p miR-18a-5p miR-199a-5p miR-19b-3p miR-210-3p miR-28b-3p miR-29a-3p miR-29c-3p miR-331-3p miR-335-5p miR-34a-5p	–	miR-139-5p miR-181d-5p miR-182-5p miR-31-5p miR-365a-3p	miR-130b-3p miR-15a-5p	miR-326	miR-128-3p miR-132-3p miR-15a-3p miR-15b-5p miR-181b-5p miR-191-5p miR-19a-3p miR-19b-3p miR-23a-3p miR-25-3b miR-27a-3p miR-30c-5p miR-574-3p miR-92a-3p	miR-130b-3p miR-17-3p miR-181a-5p miR-93-5p miR-98-5p miR-181c-5p miR-99a-5p miR-18a-5p miR-130b-5p
	Age <65 years	Rai stages 0–1	WBC <35 × 10^9^/L	B2M <3 mg/L	CD38 <30 %	*NOTCH1* wild type	del *ATM* −	*TP53* del/mut −	del *RB1* −	Trisomy 12 +	No indications for treatment
↓	miR-17-3p miR-181c-5p miR-181d-5p miR-182-5p	–	–	–	–	miR-132-3p miR-146b-5p	–	miR-155-5p	let-7b-5p let-7d-5p let-7g-5p miR-150-5p miR-155-5p miR-26b-5p miR-29b-3p miR-29c-3p	–	–

For data analysis, the Mann-Whitney *U* test was used

*↓* expression is lower in this prognostic group compared with the opposite group, *+* present, *dash* absent

CD38 expression was the factor significantly differentiating CLL patients, and 20/84 (23.8 %) analyzed miRNAs were expressed lower in the CD38+ group: let-7a-5p, miR-7c, let-7d-5p, let-7e-5p, let-7f-5p, miR-101-3p, miR-130b-3p, miR-146b-5p, miR-15a-5p, miR-182-5p, miR-18a-5p, miR-199a-5p, miR-19b-3p, miR-210-3p, miR-28b-3p, miR-29a-3p, miR-29c-3p, miR-331-3p, miR-335-5p, and miR-34a-5p (Table 4). Only one miRNA, miR182-5p, differentiated patients as ZAP70 positive and negative, being expressed at a lower level in the latter group.

Surprisingly, there were almost no differences in general miRNA expression when molecular prognostic factors were considered: miR-28b-3p was expressed lower in *IGVH*-unmutated patients, while two miRNAs miR-132-3p and miR-146-5p were expressed lower in patients with the wild type of *NOTCH1* gene (Table 4).

Considering cytogenetic prognostic factors, the one that most significantly determinated miRNA expression was trisomy 12. In patients lacking this aberration, 14 (16.66 %) miRNAs were expressed at a lower level as compared with the +12 group: miR-128-3p, miR-132-3p, miR-15a-3p, miR-15b-5p, miR-181b-5p, miR-191-5p, miR-19a-3p, miR-19b-3p, miR-23a-3p, miR-25-3b, miR-27a-3p, miR-30c-5p, miR-574-3p, and miR-92a-3p (Table 4).

*RB1* gene *locus* deletion was associated with higher expression of eight miRNAs: miR-128-3p, miR-132-3p, miR-15a-3p, miR-15b-5p, miR-181b-5p, miR-191-5p, miR-19a-3p, miR-19b-3p, miR-23a-3p, miR-25-3b, miR-27a-3p, miR-30c-5p, miR-574-3p, and miR-92a-3p. Additionally, one miRNA, miR-326, was expressed at a lower level in patients carrying *RB1* deletion. Surprisingly, the deletion of the region D13S319 localized downstream the *RB1* locus on chromosome 13 did not affect the circulating miRNAome at all. There was also no significant correlation between del13q14.3 and the expression of miR-15a-5p or miR-16-5p as assessed using Spearman’s coefficient of correlation (*r*_s_ = −0.10426 and *r*_s_ = 0.05958, respectively).

In patients with *TP53* deletion/mutation, two miRNAs were expressed lower (miR-130b-3p and miR-15a-5p) than in patients with wt*TP53*. Contrarily, in the latter group, lower expression of miR-155-5b was observed. Low expression of five miRNAs (miR-139-5p, miR-181d-5p, miR-182-5p, miR-31-5p, and miR-365a-3p) characterized patients with 11q/ATM deletion.

#### Some miRNAs are differentially expressed across several prognostic groups

Looking for differentiating miRNAs common for different prognostic groups, we have found miR-182-5p differentially expressed in four groups (patients older than 65, CD38+, ZAP70+, with del11q) (Table 5). Five miRNAs were differentially expressed in three groups: miR-28b-3p (patients with high B2M, CD38+, *IGVH*-unmutated), miR-15a-5p (patients CD38+, with *TP53* deletion/mutation, and trisomy 12), miR-125-5b and miR-99a-5p in the same prognostic groups (patients in advanced stage—Rai 2–4, patients with high B2M, patients with high WBC), and miR-132-3p (patients with high B2M, *NOTCH1* mutation and trisomy 12) (Table 5). Three other miRNAs, miR-7c, miR-15a-5p, and miR-155-5p, shared differences in expression between two prognostic groups.

**Table 5:** Differentiating miRNAs common for several prognostic groups

miRNA	No. of common groups	Age ≥65	WBC ≥35 × 10^9^/L	Rai stages 2–4	B2M ≥3 mg/L	CD38 ≥30 %	ZAP70 ≥20 %	*IGVH* unmut	*NOTCH1* mut	*TP53* mut/del	*ATM* del	*RB1* del	+12 +
miR-182-5p	4	↑	–	–	–	↓	↑	–	–	–	↓	–	–
miR-125b-5p	3	–	↓	↓	↓	–	–	–	–	–	–	–	–
miR-99a-5p	3	–	↓	↓	↓	–	–	–	–	–	–	–	–
miR-132-3p	3	–	–	–	↓	–	–	–	↑	–	–	–	↑
miR-28-3p	3	–	–	–	↓	↓	–	↓	–	–	–	–	–
miR-15a-5p	3	–	–	–	–	↓	–	–	–	↓	–	–	↑
miR-18a-5p	2	–	–	–	↓	↓	–	–	–	–	–	–	–
miR-7c	2	–	–	–	↓	↓	–	–	–	–	–	–	–
miR-155-5p	2	–	–	–	–	–	–	–	–	↑	–	↑	–

Arrows stand for statistically important differences in serum expression of miRNAs between respective groups of patients. Arrow direction points the type of the difference (lower/higher expression) between two subgroups (i.e., males/females, age ≤65 and >65, etc.). Dashes stand for no statistical differences between groups

#### The expression of miRNAs related to T and B cell differentiation and activation is associated with CLL prognostic factors

We then analyzed circulating miRNA expression regarding their functional groups with respect to B and T cell differentiation, T cell activation, and miRNAs differentially expressed in Tregs. Several functional miRNA groups were considered: (1) B cell differentiation: a. naive, b. germinal center, c. memory; (2) T cell differentiation: a. double negative (CD4−/CD8−), b. double positive (CD4+/CD8+), c. CD4+ naive, d. CD8+ naive, e. CD8+ effector, f. CD8+ memory; (3) T cell activation; and (4) Tregs. The complete list of grouped miRNAs according to manufacturer of the assay is presented in Online Resource Table S3.

Alterations in miRNA expression involved all functional groups, and most of them correlate with prognostic factors. Some of the most adverse prognostic factors in CLL (i.e., age >65 years, Rai stages 2–4, WBC >35 × 10^9^/L, CD38+ cells >30 %, B2M >3 mg/L, LDH >480 IU/L, del 11q and lack of *IGVH* mutation) were correlated with the decrease of miRNA expression (Tables 6 and 7). Only mutation of *NOTCH1* gene and male sex were found to correlate with the increase of expression of the miRNAs analyzed.

**Table 6:** The direction of statistically significant differences in serum expression of miRNA classified into functional groups depending on prognostic factors

miRNa expression—functional groups	Sex M	Age ≥65	Rai stages 2–4	WBC ≥35 × 10^9^/L	CD38 ≥30 %	ZAP70 ≥20 %	B2M ≥3 mg/L	LDH ≥480 IU/L	*IGVH* unmut	*NOTCH1* mut	del *RB1* +	del D13S319 +	*TP53* del/mut +	del *ATM* +	+12 +
B cell differentiation															
Naive	↑	↓	–	↓	↓	–	↓	↓	↓	↑	–	–	–	↓	↑
Germinal center	↑	↓	↓	↓	↓	–	↓	↓	↓	↑	–	–	↓	–	↑
Memory	–	↓	–	↓	↓	–	↓	↓	–	↑	–	↓	↑	↓	↑
T cell differentiation															
Double negative^a^	–	↓	↓	↓	↓	–	↓	↓	↓	↑	–	–	↓	↓	↑
Double positive^b^	–	–	–	–	↓	–	↓	–	↓	↑	–	–	–	↓	↑
CD4+ naive	–	–	–	↓	–	–	↓	–	–	–	–	–	–	–	↑
CD8+ naive	–	↓	–	↓	↓	–	↓	↓	↓	↑	–	–	↓↑	↓	↑
CD8+ effector	–	–	↓	–	↓	–	↓	–	–	–	–	–	–	–	↑
CD8+ memory	–	↓	–	↓	↓	–	–	–	–	–	–	–	–	–	↑
T cell activation	↑	↓	–	↓	↓	–	↓	↓	↓	↑	–	–	↓↑	↓	↑
Tregs	↑	–	–	↓	↓	–	↓	–	↓	↑	–	–	–	↓	↑

Data were analyzed using the Kruskal-Wallis test. Arrows stand for statistically important differences in serum expression of miRNAs associated with B and T cell differentiation between respective groups of patients. Arrow direction points the type of the difference (lower/higher expression) between two subgroups (i.e., males/females, age <65 and ≥65, etc.). Dashes stand for no significant differences between groups

^a^CD4−/CD8−

^b^CD4+/CD8+

**Table 7:** *P* values of the most significant differences in serum expression of miRNA classified into functional groups depending on prognostic factors

miRNa expression—functional groups	Age <65 vs ≥65	WBC <35 × 10^9^ vs ≥35 × 10^9^/L	CD38 <30 vs ≥30 %	B2M <3 vs ≥3 mg/L	LDH <480 vs ≥480 IU/L	*IGVH* mut vs unmut	*NOTCH1* mut vs unmut	*ATM* deleted vs wt	chrom 12 trisomy vs n12
B cell differentiation									
Naive	0.0018	0.0034	<0.0001	0.0008	0.0103	0.0376	0.0011	0.0130	<0.0001
Germinal center	<0.0001	<0.0001	<0.0001	<0.0001	0.0117	0.0115	0.0125	–	<0.0001
Memory	0.0005	0.0002	0.0001	0.0024	0.0440	–	0.0462	0.0111	<0.0001
T cell differentiation									
Double negative^a^	0.0008	<0.0001	<0.0001	<0.0001	0.0020	0.0050	0.0006	0.0033	<0.0001
Double positive^b^	–	–	0.0001	0.0007	–	0.0169	0.0347	0.0019	0.0006
CD4+ naive	–	0.0063	–	0.0059	–	–	–	–	0.0151
CD8+ naive	0.0052	0.0064	<0.0001	0.0008	0.0124	0.0317	0.0189	0.0081	<0.0001
CD8+ effector	–	–	0.0024	0.0009	–	–	–	–	0.0013
CD8+ memory	0.0005	0.0135	0.0007	–	–	–	–	–	<0.0001
T cell activation	<0.0001	<0.0001	<0.0001	<0.0001	0.0193	0.0051	0.0009	0.0017	<0.0001
Tregs	<0.0001	<0.0001	0.0002	0.0003	–	0.0388	0.0093	0.0006	0.0041

For data analysis, the Kruskal-Wallis test was used. Dashes stand for no statistical differences. See Table 6 for the direction of the particular difference

*mut* mutated, *unmut* unmutated, *wt* wild type, *n12* normal number of chromosomes 12

^a^CD4−/CD8−

^b^CD4+/CD8+

Status of chromosome 12 was the factor that affected the expression of miRNAs from all functional groups. In patients carrying trisomy 12, their expression was significantly higher as compared with patients without this aberration. The number of CD38-positive cells and B2M level were important as well—the expression of miRNAs from all functional groups except one (CD4+ naive and CD8+ memory associated, respectively) was significantly lower in patients with more than 30 % of CD38+ cells and high B2M (Table 6). Among well-known prognostic factors clearly associated with circulating miRNA expression, we have found also WBC, *ATM* gene status, and age—in all cases, patients classified to the adverse prognostic groups were identified with decreased miRNA expression.

Considering expression of miRNAs grouped into functional groups, *IGVH* gene status was also identified as an important differentiating factor: miRNA expression was significantly lower in unmutated patients in 7 out of 11 functional groups considered (Tables 6 and 7). As mentioned before, *NOTCH1* mutation was associated with an increased miRNA expression, and this was observed for all functional groups except CD4+ naive, CD8+ effector, and CD8+ memory T cells (Table 6).

#### The largest number of differentially expressed miRNAs between CLL prognostic groups is observed in these associated with B cell differentiation, T cell activation, and double negative T cells

Significant differences between CLL prognostic groups were found for expression of all groups of miRNAs associated with B cell differentiation: germinal center cells, naive cells, and memory B lymphocytes (Table 6). The expression of them all was clearly associated with patients’ age, WBC, CD38 status, B2M level, LDH activity, and the presence of *NOTCH1* mutation and trisomy 12 (Table 7).

Of the T cell-associated miRNAs, the most distinct expression differences involved molecules related to CD4−/CD8− cells and CD4+ naive cells as well as associated with T cell activation and differentially expressed in Tregs. Prognostic factors most important for all T-associated miRNA expression involved WBC, CD38 status, B2M level, and trisomy 12 (Tables 6 and 7).

#### Circulating miRNA expression is associated with the need of treatment initiation

We have found significant differences in the expression of some miRNAs between patients classified to the “watch and wait” group and patients with indications to treatment initiation (Table 4). Most of the differently expressed miRNAs were associated with germinal center B cells (miR-181a-5p, miR-17-3p, miR-98-5p) and T cell activation/Tregs (miR-181c-5p); their expression was lower in patients requiring treatment.

## Discussion

miRNAs are now well-known factors which may contribute to the leukemogenesis and affect the clinical course of chronic lymphocytic leukemia [1, 37]. Historically, CLL was the first among human cancers discovered to clearly associate with alterations of miRNA expression. Deletion of miR-15a and miR-16 in leukemic cells was found to result in *BCL-2* gene overexpression leading to their prolonged survival and resistance to apoptosis [16]. The exact role of miR-15/16 in CLL was reviewed in detail by Pekarsky and Croce [17]. Many studies addressed miRNA expression in lymphocytes of CLL patients and tested its correlation with clinical and prognostic parameters [38–41]. New possibilities have opened with the discovery of circulating miRNAs. Easily available from serum/plasma, urine, or saliva, they are especially convenient biomarkers in solid tumors; however, one cannot underestimate their role in leukemias. Once released from leukemic cells, miRNAs may potentially affect the cells that comprise the microenvironment and vice versa. Circulating miRNA molecules packaged into exosomes or associated with specific proteins may be engulfed by the recipient cell or may target its receptors. Both scenarios end up with the effect on recipient cell gene expression [10]. The principle of this phenomenon was described as horizontal transfer of miRNAs and is now recognized as the new form of intercellular communication [9].

The comparative study by Moussay and colleagues has shown that, like in solid tumors, the expression of most circulating miRNAs in CLL patients reflects their expression in leukemic cells [37]. It is not quite obvious, because in leukemias other blood and bone marrow cells may also impact the expression pattern. The level of circulating miRNA expression in CLL, however, is decreased as compared to miRNA expression assessed in leukemic lymphocytes; this phenomenon was also observed in our study [37].

We analyzed serum expression of selected miRNAs associated with differentiation and activation of B and T lymphocytes. Based on the assessment of 84 miRNAs, the general serum expression in CLL patients was found to be lower in comparison to normal individuals (Supplementary Table S2). Interestingly, three miRNAs of the lowest expression in our series of patients (miR-147a, miR-184, and miR-326, Table 3) are classified as molecules associated with T cell, not B cell differentiation. Expression of none of the above miRNAs correlated with prognostic factors. To date, there is no data concerning their role in CLL and neither their *loci* are associated with breakpoints typical for this disease. However, they all are thought to function as tumor suppressors (TS), at least in some cancer types [42–50].

The gene encoding miR-147a is localized at 9q33.2. It is classified to miRNAs associated with CD8 effector cells. In mice, miR-147 is induced upon stimulation of Toll-like receptors and functions as a negative regulator of inflammatory cytokine expression in macrophages [42]. Lee and colleagues reported that transfection of miR-147 into colon cancer cells (HCT116, SW480) and lung cancer cells (A-549) resulted in mesenchymal-to-epithelial transition (MET) induction and G1 cell cycle arrest. Additionally, it led to reversion of the native drug resistance of the colon cancer cell line HCT116 to gefitinib [42]. TS function of miR-147 was also described in breast cancer, where it targets EGFR-driven cell cycle network proteins and inhibits cell cycle progression and proliferation [44].

miR-184 gene *locus* was mapped to 15q25.1. Its expression is related to CD4+ naive cells. miR-184 was found to be highly expressed in umbilical cord CD4+ cells, where it inhibits NFAT1 protein synthesis thus contributing to the early adaptive immune response [45]. Zhen and coworkers reported that miR-184 targeted and inhibited *BCL2* and *CMYC* transcripts in nasopharyngeal carcinoma [46]. In neuroblastoma, miR-184 was found to target *AKT2* kinase, a member of the AKT family of proteins that are activated by the phosphatidylinositol 3′ kinase (PI3K) pathway. Ectopic upregulation of miR-184 resulted in apoptosis of neuroblastoma cells [47].

The gene for miR-326 localizes at 11q13.4. Like miR-184, miR-326 is linked to CD4+ naive cells. As a suppressor of the Hedgehog signal transduction pathway, it has been demonstrated to control the development of cerebellar neuronal progenitor and tumor cells [48]. The decreased miR-326 expression in glioma was found to be significantly associated with advanced pathological grade. The study by Kefas and colleagues showed that the expression of miR-326 in glioma inhibited NOTCH protein synthesis, but concurrently was suppressed by NOTCH, indicating a feedback loop [49]. What is more, miR-326 transfection reduced glioma cell tumorigenicity in vivo. A decrease in miR-326 expression was found in advanced breast cancer cells and irreversibly correlated with the expression of multidrug resistance associated protein 1 (MRP1) contributing to chemoresistance [50].

Considering miRNAs that were expressed on the highest level in CLL patients studied (i.e., miR-34a-5p, miR-31-5p, miR-155-5p, miR-15a-3p, and miR-29a-3p), all but one (miR-31-5p) were associated with B cell differentiation and homeostasis (Table 2, Supplementary Table S3). In several studies, their expression was shown to discriminate between CLL patients and healthy individuals, and they all have been corroborated as important for CLL development and clinical course of the disease [1, 37, 51–54]. Among others, their confirmed targets include *BCL2* (miR-34a, mir-29a, miR-15), *MCL1* (miR-15a, miR-29a), *TCL1* (miR-29a), *BAK1* (miR-150, miR-29a), *MYC* (miR-34a), *NOTCH1* and *2* (miR-34a), *CMYB* (miR-150, miR-155), *TP53INP* (miR-155), *ETS1* (miR-155), *DICER1* (miR-29a), *CDK6* (miR-29a), *SPI1* (miR-155), and *FOXP1* (miR-34a); they were reviewed by many authors [1, 37, 51, 52, 54].

The serum expression level of four of B cell-associated molecules in our study—miR-34a-5p, miR-155-5p, miR-150-5p, and miR-29a-3p—reflected their described cellular expression; yet, it was proportionally lower [39–41]. Li and colleagues reported overexpression of two miRNAs, miR-34a and miR-155, in CLL lymphocytes, and defined it as a part of activated B cell phenotype [40]. Cellular miR-34a expression in CLL is higher in patients with intact TP53 pathway and associates with longer time to treatment [55]. TP53 protein induces expression of miR-34 family (miR-34a, locus at 1p36, miR-34b/34c, locus at 11q) and provokes TP53-like responses like senescence, cell cycle arrest, and apoptosis [55]. Fabbri and coworkers described the latter two miR-34 family members as the important part of miRNA/TP53 feedback circuitry involved in CLL pathogenesis [56]. However, in our study, there was no correlation between serum miR-34a expression and *TP53* status. Tserel and coworkers described significantly increased expression of miR-34a in peripheral blood monocyte-derived macrophages [57]. Considering that nurse-like cells, present in the circulation of CLL patients, are thought to be monocyte-derived macrophages, it is possible that they are responsible for high, *TP53*-independent expression of this molecule in the serum of tested patients [58]. An important issue requiring further investigation is whether or not leukemic lymphocytes may make use of extracellular miR-34a. It is worth pointing out that miR-34a mimic, MRX34, is the first miRNA mimic currently evaluated in a multicenter phase I study in patients with hematological malignancies including CLL (ClinicalTrials.gov Identifier NCT01829971) [59].

miR-155 expression in B lymphocytes was found to discriminate between normal individuals, individuals with monoclonal B cell lymphocytosis (MBL), and CLL patients; its overexpression in the latter group is associated with aggressive disease [60, 61]. Ferrajoli and colleagues demonstrated the presence of miR-155 in circulating microvesicles of individuals with MBL and CLL, which may point to its role in microenvironmental stimuli pathway [60]. Furthermore, upregulation of miR-155 in B cells by microenvironmental signals resulted in increased sensitivity to BCR ligation [61].

Contrarily, cellular expression of miR-15a-3p in CLL is described to be low (and potentially correlated with miR-15a/miR-16-1 deletion) [16]. In our study, the mean serum miR-15a-3p expression in CLL patients was 1.433-fold of that in normal individuals (Table 2), and the same discrepancy was also noticed by Moussay and coworkers [37]. Although we observed a significant correlation between the expression of the two miRNAs from the 13q14.3 cluster, miR-15a and miR-16-5p (Spearman coefficient ratio 0.5878), there was no significant correlation between their expression and D13S319 deletion assessed by FISH. As 13q14.3 deletion is thought to be the main reason of miR-15a and miR-16-1 deficiency in CLL lymphocytes, our results may point to other blood cells as the source of serum miR-15a [17].

We confirmed the crucial role of the four discussed miRNAs in CLL by the observation that their serum expression is significantly associated with selected prognostic factors (Table 4). Lower miR-34a expression was observed in the adverse prognostic group of CD38-positive patients; patients with *TP53* deletion/mutation and *RB1* deletion were characterized by higher miR-155-5p expression. The latter group presented also with higher miR-150-5p expression. miR-15a-5p was expressed at a lower level in patients with CD38+ and those harboring 17p deletion/*TP53* mutation.

miR-31-5p was the only one of the circulating miRNAs of the highest expression a priori associated exclusively with T cell differentiation (Supplementary Table S3). Its *locus* has been mapped to chromosome 9p21.3. Aberrant expression of miR-31 was described in many solid tumors [62–64]. In some, like melanomas, its deficiency is associated with deletion, and the ectopic expression inhibits cell migration and invasion [65]. Wang and coworkers reported decreased miR-31 expression in aggressive bladder cancer [64]. On the contrary, miR-31 overexpression in colon cancer is associated with the increased proliferation potential, and in lung adenocarcinoma, it was reported to predict lymph node metastases [62, 63]. Results of our study showed that serum miR-31-p expression is lower in patients with 11q/*ATM* deletion. We have found no data about the role of miR-31 in CLL. However, some conception may come from the study by Rouas and colleagues. They have found that miR-31 negatively regulates *FOXP3* mRNA in Tregs [66]. FOXP3 is considered as master regulator of Treg development and function, and its deletions or mutations are associated with severe, even lethal autoimmune diseases [66]. CLL patients are characterized by an increased absolute number of Tregs as compared with healthy individuals. Moreover, a progressive increase of Tregs was reported in advanced stages of the disease and in patients with autoimmune cytopenias [3]. Contrasting data concern the role of Tregs in CLL: they were found to express cytolytic markers and kill autologous B cells, but may also suppress antitumor response [66]. Considering the above facts, the significance of serum miR-31 overexpression in CLL patients needs further investigation.

The analysis of the association of general serum miRNA expression with clinical, molecular, and cytogenetic prognostic factors has shown that CD38 expression discriminated the expression of 20 miRNAs (23.8 % of all analyzed). Fourteen miRNAs (16.7 %) were expressed differently depending on the status of chromosome 12; age, B2M level, and status of *RB1* gene each discriminated the expression of nine miRNAs (Table 4).

While analyzing the general miRNA expression, we were surprised to discover that neither *IGVH* gene status nor ZAP70 expression was a significant discriminating factor, as they were described to be important for cellular miRNA expression in CLL patients [37, 38, 40, 67]. ZAP70 expression status was reported by Moussay to affect also plasma miRNA expression [37]. In our study, both ZAP70 and *IGVH* status discriminated the expression of only one miRNA each: miR-182-5p (*P* = 0.0304) and miR-28b-3p (*P* = 0.0033), respectively. Serum miRNA expression may differ from lymphocyte expression pattern because miRNA molecules secreted by other blood cells bias it. Instead, the discrepancy between our results and that of Moussay et al. may be a consequence of a little different methodology: they have isolated miRNA from plasma and profiled the expression by means of Taq miRNA low-density array from Applied Biosystems [37]. Our results may be also affected in a way by the limited number of patients. Besides, as reported previously, none of the miRNA expression profiles in CLL published to date are identical—this may also reflect the heterogeneity of the disease [40].

In this study, for the first time, *NOTCH1* gene mutation was considered in miRNA profiling analysis. The role of *NOTCH1* gene mutations in CLL was reviewed by us before [27]. It is a relatively new prognostic factor correlated with shorter time-to-first treatment, which may be an important clue for clinicians [68]. When the expression of the whole pool of studied miRNAs was analyzed, only two miRNAs were found to discriminate between *NOTCH1* mutated and *NOTCH1* wild-type patients: miR-132-3p and miR-146-5p were expressed at a lower level in the latter group (Table 4). Both of them, however, are important for leukemogenesis. The recent study by Tavolaro and coworkers points to miR-132 as one of two miRNAs upregulated in CLL lymphocytes following BCR stimulation. They linked its overexpression with RB1/E2F and TP53 cascades and proproliferative effects [69]. miR-146, on the other hand, is a well-known regulator of both innate and adaptive immune responses which was implicated both in lymphomagenesis and tumor suppression. Its overexpression was reported in splenic marginal zone lymphomas and in diffuse large B cell lymphoma (DLBCL) [70].

Analysis of miRNAs whose expression differentiated patients in several prognostic groups revealed one, miR-182-5p, common for four groups (Table 5). It was also the only miRNA discriminating ZAP70+ and ZAP70− patients. To date, miR-182 was not described to be associated with CLL. However, it was found to act as an oncogene in lymphoblastic malignancies through regulation of *FOXO3A* gene, a member of forkhead transcriptional factor family, which has been implicated in tumor suppression and glucocorticoid-induced apoptosis [71].

When patients with and without indications for treatment were analyzed, a lower expression of some miRNAs in the former group was found (Table 4). Among them was miR-181a-5p, whose underexpression in CLL was described to correlate with shorter overall survival and treatment free survival [72].

The comparison of functionally grouped miRNA expression between CLL patients and healthy individuals has shown that alterations in expression pattern involve all functional groups, and most of them correlate with prognostic factors (Tables 6 and 7). This confirms the involvement of various cell populations in the pathogenesis of the disease. Three groups that were mostly associated with prognostic factors were miRNAs linked with germinal center B cells, T cell activation, and double negative lymphocytes.

Characteristic histological features of CLL are proliferation centers (PCs) located within secondary lymphatic organs, which contain prolymphocytes, paraimmunoblasts, T lymphocytes, nurse-like cells, and dendritic cells. Within proliferation centers, B cells may proliferate and undergo selection and clonal expansion in a T-dependent manner [73]. In many CLL cases, the number of proliferation centers is increased (PC-rich cases) which is associated with adverse prognosis; PCs serve as reservoir of leukemic cells and the source of minimal residual disease [41]. As the cells within PCs are more activated than circulating cells, it is quite possible that the profile of circulating miRNAs associated with cell activation will differ from the profile of peripheral blood leukemic cells.

Here we show that the level of expression of these miRNAs (especially related to germinal center B cells and T cell activation) is lower in some adverse prognostic groups: in older patients, CD38 positive, with unmutated IGVH genes, high WBC, high B2M level, and 11q/*ATM* deletion (Tables 6 and 7). The presented miRNA expression profile confirms the favorable prognostic significance of trisomy 12—in patients bearing this aberration, the expression of the discussed miRNAs was lower.

When the expression of functional miRNA groups was analyzed, *NOTCH1* status was also found to be an important discriminating factor (Tables 6 and 7). It affected the expression of all miRNAs associated with B cell differentiation and activation as well as this was linked with T cell activation, Tregs, and early stages of T cell differentiation. As *NOTCH1* activating mutations in CLL are thought to contribute to apoptosis resistance via activation of the nuclear factor-κB (NF-κB) pathway and the AKT pathway, it is quite reasonable that the status of this gene may impact miRNA and gene expression profile [74]. The expression of miRNAs analyzed in this study was higher in patients bearing *NOTCH1* mutation.

What is noteworthy, our results show that the subpopulation of Tregs seems to be equally involved in CLL development. The expression of miRNAs differently expressed in Tregs was found to be significantly associated with patients’ age, WBC, CD38 expression, and B2M level, as well as with deletion of 11q, mutational status of *NOTCH1* and *IGVH*, and trisomy 12 (Tables 6 and 7). The association of Treg number with CLL progression and coexisting immunodeficiency was reported by Lad and coworkers [3]. A recent study by Jitschin and colleagues has shed some light on the mechanisms of Treg induction. They have focused on the further component of CLL microenvironment, described as myeloid-derived suppressor cells (MDSCs) [4]. MDSCs represent a heterogeneous population of myeloid progenitors and precursors of granulocytes, macrophages, and dendritic cells [75]. MDSC differentiation is driven by CLL lymphocytes; they act by inhibiting T cell responses and promoting induction of regulatory T cells. The latter effect was linked to the fact that MDSCs produce an immunomodulatory cytokine indoleamine-2,3-dioxygenase (IDO), the first and rate-limiting enzyme of tryptophan catabolism through the kynurenine pathway [4]. However, we hypothesize that miRNA molecules circulating in peripheral blood of CLL patients may also induce Tregs.

## Conclusions

Chronic lymphocytic leukemia is a very heterogeneous disease and its pathogenesis is complex both at the molecular and the cellular level. Some pieces of the puzzle were now completed, but yet there are many to discover. Although subsequent studies on larger patient cohort are necessary, the presented results of serum miRNA expression profiling have confirmed that CLL should be considered as disease of the blood, bone marrow, and secondary lymphatic organs. Circulating miRNAs may be released by leukemic lymphocytes to tout the neighboring cells. And vice versa, all by-standing cells may potentially influence leukemic lymphocytes and help them to divide and avoid apoptosis. Thus, the studies of the mechanisms involved in CLL development, as well as the design of novel therapeutic strategies, require a holistic approach involving focus on all B and T lymphocyte populations, together with stromal cells, nurse-like cells, MDSCs, and other cells composing the microenvironment.

## Electronic supplementary material

Below is the link to the electronic supplementary material.

ESM 1 Clinical and molecular data of CLL patients (DOC 87 kb)

ESM 2 Ct values obtained in circulating miRNA expression assessment of CLL patients and healthy subjects by qT-PCR (DOC 203 kb)

ESM 3 Functional gene grouping – according to manufacturer of T-Cell & B-Cell Activation miScript miRNA PCR Array (QIAGEN) (DOCX 13 kb)

## References

[1] Bottoni A, Calin GA (2014). MicroRNAs as main players in the pathogenesis of chronic lymphocytic leukemia. MicroRNA.

[2] Keating MJ, Chiorazzi N, Messmer B, Damle RN, Allen SL, Rai KR, Ferrarini M, Kipps TJ (2003) Biology and treatment of chronic lymphocytic leukemia. Hematology Am Soc Hematol Educ Program 2003:153–175 10.1182/asheducation-2003.1.153 14633781

[3] Lad DP, Varma S, Varma N, Sachdeva MU, Bose P, Malhotra P (2015). Regulatory T-cell and T-helper 17 balance in chronic lymphocytic leukemia progression and autoimmune cytopenias. Leuk Lymphoma.

[4] Jitschin R, Braun M, Büttner M, Dettmer-Wilde K, Bricks J, Berger J, Eckart MJ, Krause SW, Oefner PJ, Le Blanc K, Mackensen A, Mougiakakos D (2014). CLL-cells induce IDOhi CD14+HLA-DRlo myeloid-derived suppressor cells that inhibit T-cell responses and promote TRegs. Blood.

[5] Lu J, Getz G, Miska EA, Alvarez-Saavedra E, Lamb J, Peck D, Sweet-Cordero A, Ebert BL, Mak RH, Ferrando AA, Downing JR, Jacks T, Horvitz HR, Golub TR (2005). MicroRNA expression profiles classify human cancers. Nature.

[6] Iorio MV, Ferracin M, Liu CG, Veronese A, Spizzo R, Sabbioni S, Magri E, Pedriali M, Fabbri M, Campiglio M, Ménard S, Palazzo JP, Rosenberg A, Musiani P, Volinia S, Nenci I, Calin GA, Querzoli P, Negrini M, Croce CM (2005). MicroRNA gene expression deregulation in human breast cancer. Cancer Res.

[7] Yanaihara N, Caplen N, Bowman E, Seike M, Kumamoto K, Yi M, Stephens RM, Okamoto A, Yokota J, Tanaka T, Calin GA, Liu CG, Croce CM, Harris CC (2006). Unique microRNA molecular profiles in lung cancer diagnosis and prognosis. Cancer Cell.

[8] Stamatopoulos B, Van Damme M, Crompot E, Dessars B, El Housni H, Mineur P, Meuleman N, Bron D, Lagneaux L (2015) Opposite prognostic significance of cellular and serum circulating microRNA-150 in chronic lymphocytic leukemia patients. Mol Med 28:123–133. doi:10.2119/molmed.2014.00214. 10.2119/molmed.2014.00214 PMC4461585 25584781

[9] Chen X, Liang H, Zhang J, Zen K, Zhang CY (2012). Secreted microRNAs: a new form of intercellular communication. Trends Cell Biol.

[10] Chen X, Liang H, Zhang J, Zen K, Zhang CY (2012). Horizontal transfer of microRNAs: molecular mechanisms and clinical applications. Protein Cell.

[11] Filip AA, Ciseł B, Wąsik-Szczepanek E (2015). Guilty bystanders: nurse-like cells as a model of microenvironmental support for leukemic lymphocytes. Clin Exp Med.

[12] Rai KR, Sawitsky A, Cronkite EP, Chanana AD, Levy RN, Pasternack BS (1975). Clinical staging of chronic lymphocytic leukemia. Blood.

[13] Binet JL, Auquier A, Dighiero G, Chastang C, Piguet H, Goasguen J, Vaugier G, Potron G, Colona P, Oberling F, Thomas M, Tchernia G, Jacquillat C, Boivin P, Lesty C, Duault MT, Monconduit M, Belabbes S, Gremy F (1981). A new prognostic classification of chronic lymphocytic leukemia derived from a multivariate survival analysis. Cancer.

[14] Döhner H, Stilgenbauer S, Döhner K, Bentz M, Lichter P (1999). Chromosome aberrations in B-cell chronic lymphocytic leukemia: reassessment based on molecular cytogenetic analysis. J Mol Med (Berl).

[15] Cotter FE, Auer RL (2007). Genetic alteration associated with chronic lymphocytic leukemia. Cytogenet Genome Res.

[16] Calin GA, Dumitru CD, Shimizu M, Bichi R, Zupo S, Noch E, Aldler H, Rattan S, Keating M, Rai K, Rassenti L, Kipps T, Negrini M, Bullrich F, Croce CM (2002). Frequent deletions and down-regulation of micro-RNA genes miR15 and miR16 at 13q14 in chronic lymphocytic leukemia. Proc Natl Acad Sci U S A.

[17] Pekarsky Y, Croce CM (2015). Role of miR-15/16 in CLL. Cell Death Differ.

[18] Zenz T, Kröber A, Scherer K, Häbe S, Bühler A, Benner A, Denzel T, Winkler D, Edelmann J, Schwänen C, Döhner H, Stilgenbauer S (2008). Monoallelic TP53 inactivation is associated with poor prognosis in chronic lymphocytic leukemia: results from a detailed genetic characterization with long-term follow-up. Blood.

[19] Döhner H, Stilgenbauer S, Benner A, Leupolt E, Kröber A, Bullinger L, Döhner K, Bentz M, Lichter P (2000). Genomic aberrations and survival in chronic lymphocytic leukemia. N Engl J Med.

[20] Damle RN, Wasil T, Fais F, Ghiotto F, Valetto A, Allen SL, Buchbinder A, Budman D, Dittmar K, Kolitz J, Lichtman SM, Schulman P, Vinciguerra VP, Rai KR, Ferrarini M, Chiorazzi N (1999). Ig V gene mutation status and CD38 expression as novel prognostic indicators in chronic lymphocytic leukemia. Blood.

[21] Orchard JA, Ibbotson RE, Davis Z, Wiestner A, Rosenwald A, Thomas PW, Hamblin TJ, Staudt LM, Oscier DG (2004). ZAP-70 expression and prognosis in chronic lymphocytic leukaemia. Lancet.

[22] Rassenti LZ, Huynh L, Toy TL, Chen L, Keating MJ, Gribben JG, Neuberg DS, Flinn IW, Rai KR, Byrd JC, Kay NE, Greaves A, Weiss A, Kipps TJ (2004). ZAP-70 compared with immunoglobulin heavy-chain gene mutation status as a predictor of disease progression in chronic lymphocytic leukemia. N Engl J Med.

[23] Puente XS, Pinyol M, Quesada V, Conde L, Ordóñez GR, Villamor N, Escaramis G, Jares P, Beà SS, González-Díaz M, Bassaganyas L, Baumann T, Juan M, López-Guerra M, Colomer D, Tubío JM, López C, Navarro A, Tornador C, Aymerich M, Rozman M, Hernández JM, Puente DA, Freije JM, Velasco G, Gutiérrez-Fernández A, Costa D, Carrió A, Guijarro S, Enjuanes A, Hernández L, Yagüe J, Nicolás P, Romeo-Casabona CM, Himmelbauer H, Castillo E, Dohm JC, de Sanjosé S, Piris MA, de Alava E, San Miguel J, Royo R, Gelpí JL, Torrents D, Orozco M, Pisano DG, Valencia A, Guigó R, Bayés M, Heath S, Gut M, Klatt P, Marshall J, Raine K, Stebbings LA, Futreal PA, Stratton MR, Campbell PJ, Gut I, López-Guillermo A, Estivill X, Montserrat E, López-Otín C, Campo E (2011). Whole-genome sequencing identifies recurrent mutations in chronic lymphocytic leukaemia. Nature.

[24] Rossi D, Bruscaggin A, Spina V, Rasi S, Khiabanian H, Messina M, Fangazio M, Vaisitti T, Monti S, Chiaretti S, Guarini A, Del Giudice I, Cerri M, Cresta S, Deambrogi C, Gargiulo E, Gattei V, Forconi F, Bertoni F, Deaglio S, Rabadan R, Pasqualucci L, Foà R, Dalla-Favera R, Gaidano G (2011). Mutations of the SF3B1 splicing factor in chronic lymphocytic leukemia: association with progression and fludarabine-refractoriness. Blood.

[25] Rossi D, Fangazio M, Rasi S, Vaisitti T, Monti S, Cresta S, Chiaretti S, Del Giudice I, Fabbri G, Bruscaggin A, Spina V, Deambrogi C, Marinelli M, Famà R, Greco M, Daniele G, Forconi F, Gattei V, Bertoni F, Deaglio S, Pasqualucci L, Guarini A, Dalla-Favera R, Foà R, Gaidano G (2012). Disruption of BIRC3 associates with fludarabine chemorefractoriness in TP53 wild-type chronic lymphocytic leukemia. Blood.

[26] Rossi D, Rasi S, Spina V, Bruscaggin A, Monti S, Ciardullo C, Deambrogi C, Khiabanian H, Serra R, Bertoni F, Forconi F, Laurenti L, Marasca R, Dal-Bo M, Rossi FM, Bulian P, Nomdedeu J, Del Poeta G, Gattei V, Pasqualucci L, Rabadan R, Foà R, Dalla-Favera R, Gaidano G (2013). Integrated mutational and cytogenetic analysis identifies new prognostic subgroups in chronic lymphocytic leukemia. Blood.

[27] Filip AA (2013). New boys in town: prognostic role of SF3B1, NOTCH1 and other cryptic alterations in chronic lymphocytic leukemia and how it works. Leuk Lymphoma.

[28] Balatti V, Bottoni A, Palamarchuk A, Alder H, Rassenti LZ, Kipps TJ, Pekarsky Y, Croce CM (2012). NOTCH1 mutations in CLL associated with trisomy 12. Blood.

[29] International CLL-IPI working group (2016). An international prognostic index for patients with chronic lymphocytic leukaemia (CLL-IPI): a meta-analysis of individual patient data. Lancet Oncol.

[30] Pekarsky Y, Santanam U, Cimmino A, Palamarchuk A, Efanov A, Maximov V, Volinia S, Alder H, Liu GC, Rassenti L, Calin GA, Hagan JP, Kipps T, Croce CM (2006). Tcl1 expression in chronic lymphocytic leukemia is regulated by miR-29 and miR-181. Cancer Res.

[31] Visone R, Rassenti LZ, Veronese A, Taccioli C, Costinean S, Aguda BD, Volinia S, Ferracin M, Palatini J, Balatti V, Alder H, Negrini M, Kipps TJ, Croce CM (2009). Karyotype-specific microRNA signature in chronic lymphocytic leukemia. Blood.

[32] http://p53.iarc.fr/Download/TP53_DirectSequencing_IARC.pdf. Assessed 15 July 2016

[33] Zenz T, Vollmer D, Trbusek M, Smardova J, Benner A, Soussi T, Helfrich H, Heuberger M, Hoth P, Fuge M, Denzel T, Häbe S, Malcikova J, Kuglik P, Truong S, Patten N, Wu L, Oscier D, Ibbotson R, Gardiner A, Tracy I, Lin K, Pettitt A, Pospisilova S, Mayer J, Hallek M, Döhner H, Stilgenbauer S (2010). European Research Initiative on CLL (ERIC). TP53 mutation profile in chronic lymphocytic leukemia: evidence for a disease specific profile from a comprehensive analysis of 268 mutations. Leukemia.

[34] van Dongen JJ, Langerak AW, Brüggemann M, Evans PA, Hummel M, Lavender FL, Delabesse E, Davi F, Schuuring E, García-Sanz R, van Krieken JH, Droese J, González D, Bastard C, White HE, Spaargaren M, González M, Parreira A, Smith JL, Morgan GJ, Kneba M, Macintyre EA (2003). Design and standardization of PCR primers and protocols for detection of clonal immunoglobulin and T-cell receptor gene recombinations in suspect lymphoproliferations: report of the BIOMED-2 Concerted Action BMH4-CT98–3936. Leukemia.

[35] Livak KJ, Schmittgen TD (2001). Analysis of relative gene expression data using real-time quantitative PCR and the 2-ΔΔCt method. Methods.

[36] Ouillette P, Collins R, Shakhan S, Li J, Li C, Shedden K, Malek SN (2011). The prognostic significance of various 13q14 deletions in chronic lymphocytic leukemia. Clin Cancer Res.

[37] Moussay E, Wang K, Cho JH, van Moer K, Pierson S, Paggetti J, Nazarov PV, Palissot V, Hood LE, Berchem G, Galas DJ (2011). MicroRNA as biomarkers and regulators in B-cell chronic lymphocytic leukemia. Proc Natl Acad Sci U S A.

[38] Negrini M, Cutrona G, Bassi C, Fabris S, Zagatti B, Colombo M, Ferracin M, D’Abundo L, Saccenti E, Matis S, Lionetti M, Agnelli L, Gentile M, Recchia AG, Bossio S, Reverberi D, Rigolin G, Calin GA, Sabbioni S, Russo G, Tassone P, Morabito F, Ferrarini M, Neri A (2014). MicroRNAome expression in chronic lymphocytic leukemia: comparison with normal B-cell subsets and correlations with prognostic and clinical parameters. Clin Cancer Res.

[39] Zanette DL, Rivadavia F, Molfetta GA, Barbuzano FG, Proto-Siqueira R, Silva-Jr WA, Falcão RP, Zago MA (2007). miRNA expression profiles in chronic lymphocytic and acute lymphocytic leukemia. Braz J Med Biol Res.

[40] Li S, Moffett HF, Lu J, Werner L, Zhang H, Ritz J, Neuberg D, Wucherpfennig KW, Brown JR, Novina CD (2011). MicroRNA expression profiling identifies activated B cell status in chronic lymphocytic leukemia cells. PLoS One.

[41] Wang M, Tan LP, Dijkstra MK, van Lom K, Robertus JL, Harms G, Blokzijl T, Kooistra K, van T’veer MB, Rosati S, Visser L, Jongen-Lavrencic M, Kluin PM, van den Berg A (2008). miRNA analysis in B-cell chronic lymphocytic leukaemia: proliferation centres characterized by low miR-150 and high BIC/miR-155 expression. J Pathol.

[42] Lee CG, McCarthy S, Gruidl M, Timme C, Yeatman TJ (2014) MicroRNA-147 induces a mesenchymal-to-epithelial transition (MET) and reverses EGFR inhibitor resistance. PLoS One 9:e84597. doi:10.1371/journal.pone.0084597. eCollection 2014. 10.1371/journal.pone.0084597 PMC3893127 24454732

[43] Liu G, Friggeri A, Yang Y, Park YJ, Tsuruta Y, Abraham E (2009). miR-147, a microRNA that is induced upon Toll-like receptor stimulation, regulates murine macrophage inflammatory responses. Proc Natl Acad Sci U S A.

[44] Uhlmann S, Mannsperger H, Zhang JD, Horvat EÁ, Schmidt C, Küblbeck M, Henjes F, Ward A, Tschulena U, Zweig K, Korf U, Wiemann S, Sahin O (2012). Global microRNA level regulation of EGFR-driven cell-cycle protein network in breast cancer. Mol Syst Biol.

[45] Weitzel RP, Lesniewski ML, Haviernik P, Kadereit S, Leahy P, Greco NJ, Laughlin MJ (2009). microRNA 184 regulates expression of NFAT1 in umbilical cord blood CD4+ T cells. Blood.

[46] Zhen Y, Liu Z, Yang H, Yu X, Wu Q, Hua S, Long X, Jiang Q, Song Y, Cheng C, Wang H, Zhao M, Fu Q, Lyu X, Chen Y, Fan Y, Liu Y, Li X, Fang W (2013). Tumor suppressor PDCD4 modulates miR-184-mediated direct suppression of C-MYC and BCL2 blocking cell growth and survival in nasopharyngeal carcinoma. Cell Death Dis.

[47] Foley NH, Bray IM, Tivnan A, Bryan K, Murphy DM, Buckley PG, Ryan J, O’Meara A, O’Sullivan M, Stallings RL (2010). MicroRNA-184 inhibits neuroblastoma cell survival through targeting the serine/threonine kinase AKT2. Mol Cancer.

[48] Wang S, Lu S, Geng S, Ma S, Liang Z, Jiao B (2013). Expression and clinical significance of microRNA-326 in human glioma miR-326 expression in glioma. Med Oncol.

[49] Kefas B, Comeau L, Floyd DH, Seleverstov O, Godlewski J, Schmittgen T, Jiang J, DiPierro CG, Li Y, Chiocca EA, Lee J, Fine H, Abounader R, Lawler S, Purow B (2009). The neuronal microRNA miR-326 acts in a feedback loop with notch and has therapeutic potential against brain tumors. J Neurosci.

[50] Liang Z, Wu H, Xia J, Li Y, Zhang Y, Huang K, Wagar N, Yoon Y, Cho HT, Scala S, Shim H (2010). Involvement of miR-326 in chemotherapy resistance of breast cancer through modulating expression of multidrug resistance-associated protein 1. Biochem Pharmacol.

[51] Danger R, Braza F, Giral M, Soulillou JP, Brouard S (2014) MicroRNAs, major players in B cells homeostasis and function. Front Immunol 5:98. doi:10.3389/fimmu.2014.00098. eCollection 2014 10.3389/fimmu.2014.00098 PMC3949129 24653724

[52] Lawrie CH (2013). MicroRNAs and lymphomagenesis: a functional review. Br J Haematol.

[53] de Yébenes VG, Bartolomé-Izquierdo N, Ramiro AR (2013). Regulation of B-cell development and function by microRNAs. Immunol Rev.

[54] Mraz M, Kipps TJ (2013). MicroRNAs and B cell receptor signaling in chronic lymphocytic leukemia. Leuk Lymphoma.

[55] Merkel O, Asslaber D, Piñón JD, Egle A, Greil R (2010). Interdependent regulation of p53 and miR-34a in chronic lymphocytic leukemia. Cell Cycle.

[56] Fabbri M, Bottoni A, Shimizu M, Spizzo R, Nicoloso MS, Rossi S, Barbarotto E, Cimmino A, Adair B, Wojcik SE, Valeri N, Calore F, Sampath D, Fanini F, Vannini I, Musuraca G, Dell’Aquila M, Alder H, Davuluri RV, Rassenti LZ, Negrini M, Nakamura T, Amadori D, Kay NE, Rai KR, Keating MJ, Kipps TJ, Calin GA, Croce CM (2011). Association of a microRNA/TP53 feedback circuitry with pathogenesis and outcome of B-cell chronic lymphocytic leukemia. JAMA.

[57] Tserel L, Runnel T, Kisand K, Pihlap M, Bakhoff L, Kolde R, Peterson H, Vilo J, Peterson P, Rebane A (2011). MicroRNA expression profiles of human blood monocyte-derived dendritic cells and macrophages reveal miR-511 as putative positive regulator of Toll-like receptor 4. J Biol Chem.

[58] Filip AA, Ciseł B, Koczkodaj D, Wąsik-Szczepanek E, Piersiak T, Dmoszyńska A (2013). Circulating microenvironment of CLL: are nurse-like cells related to tumor-associated macrophages?. Blood Cells Mol Dis.

[59] van Roosbroeck K, Calin GA (2016). MicroRNAs in chronic lymphocytic leukemia: miRacle or miRage for prognosis and targeted therapies?. Semin Oncol.

[60] Ferrajoli A, Shanafelt TD, Ivan C, Shimizu M, Rabe KG, Nouraee N, Ikuo M, Ghosh AK, Lerner S, Rassenti LZ, Xiao L, Hu J, Reuben JM, Calin S, You MJ, Manning JT, Wierda WG, Estrov Z, O’Brien S, Kipps TJ, Keating MJ, Kay NE, Calin GA (2013). Prognostic value of miR-155 in individuals with monoclonal B-cell lymphocytosis and patients with B chronic lymphocytic leukemia. Blood.

[61] Cui B, Chen L, Zhang S, Mraz M, Fecteau JF, Yu J, Ghia EM, Zhang L, Bao L, Rassenti LZ, Messer K, Calin GA, Croce KM, Kipps TJ (2014). MicroRNA-155 influences B-cell receptor signaling and associates with aggressive disease in chronic lymphocytic leukemia. Blood.

[62] Meng W, Ye Z, Cui R, Perry J, Dedousi-Huebner V, Huebner A, Wang Y, Li B, Volinia S, Nakanishi H, Kim T, Suh SS, Ayers LW, Ross P, Croce CM, Chakravarti A, Jin VX, Lautenschlaeger T (2013). MicroRNA-31 predicts the presence of lymph node metastases and survival in patients with lung adenocarcinoma. Clin Cancer Res.

[63] Xu RS, Wu XD, Zhang SQ, Li CF, Yang L, Li DD, Zhang BG, Zhang Y, Jin JP, Zhang B (2013). The tumor suppressor gene RhoBTB1 is a novel target of miR-31 in human colon cancer. Int J Oncol.

[64] Wang S, Li Q, Wang K, Dai Y, Yang J, Xue S, Han F, Zhang Q, Liu J, Wu W (2013). Decreased expression of microRNA-31 associates with aggressive tumor progression and poor prognosis in patients with bladder cancer. Clin Transl Oncol.

[65] Asangani IA, Harms PW, Dodson L, Pandhi M, Kunju LP, Maher CA, Fullen DR, Johnson TM, Giordano TJ, Palanisamy N, Chinnaiyan AM (2012). Genetic and epigenetic loss of microRNA-31 leads to feed-forward expression of EZH2 in melanoma. Oncotarget.

[66] Rouas R, Fayyad-Kazan H, El Zein N, Lewalle P, Rothé F, Simion A, Akl H, Mourtada M, El Rifai M, Burny A, Romero P, Martiat P, Badran B (2009). Human natural Treg microRNA signature: role of microRNA-31 and microRNA-21 in FOXP3 expression. Eur J Immunol.

[67] Calin GA, Ferracin M, Cimmino A, Di Leva G, Shimizu M, Wojcik SE, Iorio MV, Visone R, Sever NI, Fabbri M, Iuliano R, Palumbo T, Pichiorri F, Roldo C, Garzon R, Sevignani C, Rassenti L, Alder H, Volinia S, Liu CG, Kipps TJ, Negrini M, Croce CM (2005). A microRNA signature associated with prognosis and progression in chronic lymphocytic leukemia. N Engl J Med.

[68] Baliakas P, Hadzidimitriou A, Sutton LA, Rossi D, Minga E, Villamor N, Larrayoz M, Kminkova J, Agathangelidis A, Davis Z, Tausch E, Stalika E, Kantorova B, Mansouri L, Scarfò L, Cortese D, Navrkalova V, Rose-Zerilli MJ, Smedby KE, Juliusson G, Anagnostopoulos A, Makris AM, Navarro A, Delgado J, Oscier D, Belessi C, Stilgenbauer S, Ghia P, Pospisilova S, Gaidano G, Campo E, Strefford JC, Stamatopoulos K, Rosenquist R (2015). European Research Initiative on CLL (ERIC). Recurrent mutations refine prognosis in chronic lymphocytic leukemia. Leukemia.

[69] Tavolaro S, Colombo T, Chiaretti S, Peragine N, Fulci V, Ricciardi MR, Messina M, Bonina S, Brugnoletti F, Marinelli M, di Maio V, Mauro FR, del Giudice I, Macino G, Foà R, Guarini A (2015). Increased chronic lymphocytic leukemia proliferation upon IgM stimulation is sustained by the upregulation of miR-132 and miR-212. Genes Chromosomes Cancer.

[70] Szenthe K, Koroknai A, Banati F, Bathori Z, Lozsa R, Burgyan J, Wolf H, Salamon D, Nagy K, Niller HH, Minarovits J (2013). The 5′ regulatory sequences of active miR-146a promoters are hypomethylated and associated with euchromatic histone modification marks in B lymphoid cells. Biochem Biophys Res Commun.

[71] Yang A, Ma J, Wu M, Qin W, Zhao B, Shi Y, Jin Y, Xie Y (2012). Aberrant microRNA-182 expression is associated with glucocorticoid resistance in lymphoblastic malignancies. Leuk Lymphoma.

[72] Zhu DX, Zhu W, Fang C, Fan L, Zou ZJ, Wang YH, Liu P, Hong M, Miao KR, Liu P, Xu W, Li JY (2012). miR-181a/b significantly enhances drug sensitivity in chronic lymphocytic leukemia cells via targeting multiple anti-apoptosis genes. Carcinogenesis.

[73] Rossi M, Fuligni F, Ciccone M, Agostinelli C, Righi S, Luciani M, Laginestra MA, Rigolin GM, Sapienza MR, Gazzola A, Mannu C, Cuneo A, Pileri S, Piccaluga PP (2013). Hsa-miR-15a and Hsa-miR-16-1 expression is not related to proliferation centers abundance and other prognostic factors in chronic lymphocytic leukemia. Biomed Res Int.

[74] Wickremasinghe RG, Prentice AG, Steele AJ (2011). p53 and Notch signaling in chronic lymphocytic leukemia: clues to identifying novel therapeutic strategies. Leukemia.

[75] Zirlik K (2014). MDSCs: the final frontier of the microenvironment in CLL?. Blood.

