# Supplementary material for: Expression of circulating miRNAs associated with lymphocyte differentiation and activation in CLL—another piece in the puzzle
Source: Ann Hematol. 2016 Oct 12;96(1):33–50. doi: 10.1007/s00277-016-2840-6 (PMC5203831; doi:10.1007/s00277-016-2840-6)
Supplement: Supplementary file 1 — Clinical and molecular data of CLL patients (DOC 87 kb) [file 277_2016_2840_MOESM1_ESM.doc]

**Expression of circulating miRNAs associated with lymphocyte differentiation and activation in CLL – another piece in the puzzle.**

Annals of Hematology.

Agata A. Filip^1^, Anna Grenda^1^, Sylwia Popek^1^, Dorota Koczkodaj^1^, Małgorzata Michalak-Wojnowska^1^, Michał Budzyński^1^, Ewa Wąsik-Szczepanek^2^, Szymon Zmorzyński^1^, Agnieszka Karczmarczyk^3^, Krzysztof Giannopoulos^3^.

1. Department of Cancer Genetics, Medical University of Lublin, Poland

2. Department of Hematooncology and Bone Marrow Transplantation, Medical University of Lublin, Poland

3. Department of Experimental Hematooncology, Medical University of Lublin, Poland

Correspondence to: Agata A. Filip, Department of Cancer Genetics, Medical University of Lublin, Radziwiłłowska 11, 20-080 Lublin, Poland. Tel/fax: +48 81 4486100, e-mail: [aafilip@hotmail.com](mailto:aafilip@hotmail.com)

**Table S1.**

**Clinical and molecular data of CLL patients**

| **Pt #** | **sex** | **age** | **Rai**  **stage** | **WBC**  **(x10^9^/L)** | **Lymph**  **(x10^9^/L)** | **CD5**  **%** | **CD19**  **%** | **CD23**  **%** | **CD38**  **%** | **PLT**  **(x10^9^/L)** | **B2M**  **mg/l** | **LDH**  **IU/L** | **ZAP70**  **%** | ***IGVH***  **mut** | ***NOTCH1***  **mut** | ***RB1***  **%** | **D13S319**  **%** | ***TP53***  **del/mut** | ***ATM***  **%** | **+12**  **%** |
| --- | --- | --- | --- | --- | --- | --- | --- | --- | --- | --- | --- | --- | --- | --- | --- | --- | --- | --- | --- | --- |
| **1** | F | 60 | 0 | 14.29 | 7.06 | 55.59 | 57.48 | 56.45 | 51.96 | 255 | ND | 378 | 6.9 | no | no | 4.3 | 5.1 | yes | 15 | 9 |
| **2** | M | 62 | 4 | 742 | 721 | 85.04 | 95.71 | 76.6 | 53.96 | 128 | 5.55 | 325 | 13.45 | no | yes | 7.1 | 1.4 | yes | 20 | 0 |
| **3** | F | 54 | 1 | 20.17 | 14.41 | 79.62 | 76.19 | 71.89 | 0.62 | 170 | 2.85 | 346 | 19.96 | no | no | 9.4 | 45.7 | yes | 28.3 | 0 |
| **4** | F | 62 | 1 | 17.33 | 12.64 | 75.62 | 81.81 | 75.96 | 45.69 | 221 | 2.21 | 360 | 6.06 | no | no | 9 | 34.1 | no | 73 | 0 |
| **5** | F | 67 | 2 | 18.7 | 13.55 | 53.67 | 67.89 | 45.22 | 33.58 | 220 | 4.47 | 681 | 8.52 | no | no | 3.3 | 6.5 | yes | 1.4 | 4.7 |
| **6** | F | 85 | 0 | 14.47 | 10.18 | 72.54 | 73.5 | 72.11 | 26.66 | 155 | 1.99 | 407 | 33.28 | no | yes | 0.9 | 8.1 | yes | 2.9 | 11.1 |
| **7** | M | 64 | 2 | 35.4 | 22.58 | 77.92 | 80.31 | 63.1 | 67.61 | 212 | 3.02 | 579 | 42.68 | no | yes | 9.5 | 20.2 | no | 4.3 | 7.4 |
| **8** | M | 76 | 0 | 37.1 | 30.09 | 93.03 | 95.98 | 92.74 | 14.08 | 177 | 2.13 | 316 | 20.05 | no | yes | 3.7 | 50 | no | 1.9 | 1 |
| **9** | F | 67 | 1 | 68.73 | 45.06 | 83.7 | 89.14 | 71.08 | 50.72 | 187 | 3.67 | 442 | 27.03 | no | no | 6.5 | 0.9 | no | 5.5 | 0 |
| **10** | M | 70 | 2 | 36.05 | 28.72 | 49.74 | 84.84 | 79.28 | 15.02 | 325 | 7.02 | ND | 34.68 | no | no | 2 | 10.5 | yes | 48.5 | 0 |
| **11** | M | 50 | 1 | 17.97 | 13.05 | 76.89 | 82.02 | 70.3 | 4.04 | 211 | 2.21 | 372 | 15.39 | yes | no | 14 | 16 | no | 21 | 5 |
| **12** | F | 64 | 0 | 41.26 | 35.39 | 89.45 | 90.59 | 89.26 | 6.64 | 191 | 3.17 | 402 | 3.17 | no | no | 1.8 | 66.9 | no | 10.9 | 0 |
| **13** | M | 58 | 0 | 20.75 | 13.1 | 78.59 | 81.14 | 77.57 | 23.9 | 214 | 1.39 | 364 | 25.9 | yes | no | 2.5 | 7.3 | no | 1.5 | 0 |
| **14** | M | 59 | 1 | 25.98 | 19.15 | 75.29 | 85.88 | 85 | 6.52 | 269 | ND | ND | 8.66 | yes | no | 8.5 | 22 | no | 3.9 | 0 |
| **15** | F | 62 | 1 | 41.9 | 28.32 | 72.97 | 74.32 | 72.99 | 10.52 | 201 | 2.54 | 434 | 22.92 | no | no | 40 | 1.4 | yes | 3.3 | 4.6 |
| **16** | F | 79 | 2 | 110 | 100.97 | 91.41 | 93.56 | 31.5 | 0.37 | 208 | 3.14 | 462 | 4.19 | yes | no | 3.8 | 78 | yes | 0.4 | 0 |
| **17** | M | 64 | 2 | 90.11 | 87 | 61.73 | 81.09 | 76.15 | 55.58 | 151 | 9.31 | 499 | 21.19 | no | no | 10.7 | 40 | yes | 22 | 0 |
| **18** | F | 71 | 0 | 32.63 | 29.95 | 67.78 | 87.99 | 88.02 | 1.08 | 168 | 2.36 | 985 | 3.97 | yes | no | 9 | 18.4 | no | 6.1 | 0 |
| **19** | M | 59 | 2 | 41.12 | 38.2 | 87.65 | 91.23 | 89.19 | 8.25 | 373 | ND | ND | 7.14 | ND | no | 62.2 | 15 | yes | 0.4 | 0 |
| **20** | M | 74 | 0 | 18.7 | 12.7 | ND | ND | ND | 12.8 | 187 | ND | ND | 8.71 | no | yes | 1.6 | 6.8 | no | 8.5 | 6.1 |
| **21** | M | 73 | 3 | 106.8 | 97 | 5.36 | 65.28 | 68.79 | 0.54 | 124 | 10.5 | 687 | 2.61 | yes | no | 6.5 | 85 | yes | 0.9 | 23.2 |
| **22** | M | 64 | 1 | 56.84 | 41.84 | 3.87 | 75.47 | 74.79 | 9.87 | 197 | 5.19 | 379 | 7.67 | no | no | 2.3 | 9.1 | no | 0.5 | 0 |

WBC - white blood cells count, Lymph - absolute lymphocyte count, PLT - absolute platelet count, B2M - beta-2-microglobuline, LDH - lactate dehydrogenase,

mut – mutation, del - deletion, ND - not determined
