# Supplementary material for: Expression of circulating miRNAs associated with lymphocyte differentiation and activation in CLL—another piece in the puzzle
Source: Ann Hematol. 2016 Oct 12;96(1):33–50. doi: 10.1007/s00277-016-2840-6 (PMC5203831; doi:10.1007/s00277-016-2840-6)
Supplement: Supplementary file 2 — Ct values obtained in circulating miRNA expression assessment of CLL patients and healthy subjects by qT-PCR (DOC 203 kb) [file 277_2016_2840_MOESM2_ESM.doc]

**Expression of circulating miRNAs associated with lymphocyte differentiation and activation in CLL – another piece in the puzzle.**

Annals of Hematology.

Agata A. Filip^1^, Anna Grenda^1^, Sylwia Popek^1^, Dorota Koczkodaj^1^, Małgorzata Michalak-Wojnowska^1^, Michał Budzyński^1^, Ewa Wąsik-Szczepanek^2^, Szymon Zmorzyński^1^, Agnieszka Karczmarczyk^3^, Krzysztof Giannopoulos^3^.

1. Department of Cancer Genetics, Medical University of Lublin, Poland

2. Department of Hematooncology and Bone Marrow Transplantation, Medical University of Lublin, Poland

3. Department of Experimental Hematooncology, Medical University of Lublin, Poland

Correspondence to: Agata A. Filip, Department of Cancer Genetics, Medical University of Lublin, Radziwiłłowska 11, 20-080 Lublin, Poland. Tel/fax: +48 81 4486100, e-mail: aafilip@hotmail.com

**Table S2.**

**Ct values obtained in circulating miRNA expression assessment of CLL patients and healthy subjects by qT-PCR.**

| **miRNA/patient** | **1** | **2** | **3** | **4** | **5** | **6** | **7** | **8** | **9** | **10** | **11** | **12** | **13** | **14** | **15** | **16** | **17** | **18** | **19** | **20** | **21** | **22** | **Cal** |
| --- | --- | --- | --- | --- | --- | --- | --- | --- | --- | --- | --- | --- | --- | --- | --- | --- | --- | --- | --- | --- | --- | --- | --- |
| **let-7a-5p** | 30.0595 | 24.1564 | 24.9446 | 26.0249 | 26.4882 | 27.8566 | 24.7870 | 28.34399 | 26.2603 | 26.7113 | 22.8115 | 23.4363 | 24.3397 | 24.8480 | 24.9293 | 26.6931 | 22.6576 | 14.1929 | 24.9523 | 26.0859 | 27.7101 | 22.8343 | 24.9725 |
| **let-7b-5p** | 29.2809 | 23.3522 | 24.2607 | 25.4567 | 25.7223 | 27.7416 | 25.3418 | 29.31673 | 25.3443 | 25.6494 | 22.4883 | 22.8325 | 24.5676 | 25.7995 | 24.7682 | 27.3855 | 21.9056 | 15.6753 | 24.8008 | 25.7408 | 27.7543 | 23.4174 | 25.3371 |
| **miR-7c** | 32.2442 | 25.9996 | 26.6831 | 28.2064 | 28.6026 | 30.0719 | 28.3307 | 31.17794 | 28.4071 | 28.9375 | 25.4444 | 25.8984 | 27.0392 | 28.2687 | 27.8499 | 29.8881 | 24.8700 | 14.2097 | 27.9378 | 28.7560 | 30.5483 | 23.5298 | 27.6168 |
| **let-7d-5p** | 30.6494 | 27.0018 | 27.1536 | 27.8712 | 29.1718 | 29.7979 | 26.9287 | 30.75036 | 27.9387 | 28.4258 | 24.5410 | 25.1000 | 26.1588 | 26.3786 | 27.3434 | 28.6920 | 24.4865 | 15.7182 | 26.9143 | 27.8235 | 29.9238 | 23.5180 | 26.5175 |
| **let-7e-5p** | 32.2554 | 26.6947 | 27.7307 | 28.7741 | 28.8642 | 30.5980 | 27.5650 | 31.77641 | 29.1324 | 29.4282 | 25.7093 | 26.4245 | 26.8557 | 27.8286 | 28.1495 | 29.1638 | 25.6708 | 14.2291 | 27.6607 | 28.5943 | 29.7318 | 23.4648 | 27.5048 |
| **let-7f-5p** | 31.4179 | 25.6700 | 26.4193 | 27.5100 | 28.4043 | 29.1680 | 26.2674 | 29.81743 | 27.9637 | 28.3408 | 24.1195 | 25.4153 | 25.3826 | 25.6825 | 26.7834 | 27.3971 | 23.9581 | 14.2084 | 26.1218 | 27.2934 | 29.2784 | 22.9321 | 26.2558 |
| **let-7g-5p** | 29.6410 | 26.3849 | 26.4747 | 27.1742 | 29.3292 | 29.8908 | 26.0027 | 31.43459 | 27.9366 | 27.7580 | 23.4617 | 25.4363 | 25.6733 | 26.2397 | 26.6959 | 27.0242 | 22.8970 | 15.6942 | 26.7055 | 27.7363 | 29.2320 | 23.6233 | 26.7545 |
| **let-7i-5p** | 29.9182 | 26.6387 | 25.0016 | 27.2375 | 28.9724 | 29.9760 | 25.1617 | 33.40435 | 27.3615 | 25.9594 | 23.7185 | 23.1357 | 24.6314 | 25.4468 | 28.2259 | 29.4522 | 23.5484 | 14.1824 | 26.4840 | 27.5552 | 28.3585 | 23.7690 | 26.7626 |
| **miR-100-5p** | 30.8965 | 27.5669 | 29.3881 | 29.4866 | 30.8177 | 31.5883 | 29.3243 | 33.22336 | 29.7180 | 29.9638 | 29.7300 | 28.8130 | 28.5660 | 29.4457 | 30.6529 | 30.3621 | 29.5417 | 14.2719 | 29.5497 | 29.6325 | 30.8512 | 26.8562 | 29.6926 |
| **miR-101-3p** | 28.9389 | 30.5352 | 25.9350 | 27.4598 | 30.2489 | 31.6859 | 25.9396 | 28.73078 | 28.1199 | 24.9476 | 23.6769 | 22.8280 | 25.1458 | 25.6992 | 28.2982 | 28.9752 | 24.4254 | 15.6782 | 27.3914 | 28.2945 | 28.8867 | 23.8047 | 26.9047 |
| **miR-106b-5p** | 28.0165 | 28.7282 | 24.5207 | 26.7299 | 29.4643 | 30.2448 | 24.8494 | 32.54678 | 27.6849 | 25.0723 | 23.7583 | 21.6446 | 23.6075 | 24.7771 | 29.2830 | 30.2790 | 23.8997 | 14.1931 | 27.3189 | 27.6870 | 29.3913 | 23.3948 | 26.4530 |
| **miR-125b-5p** | 30.4477 | 31.5063 | 27.6419 | 29.4713 | 31.4549 | 31.5806 | 30.6394 | 34.46188 | 30.1772 | 31.4694 | 29.9025 | 28.5164 | 28.1690 | 28.8845 | 32.7049 | 33.0951 | 30.1966 | 14.2622 | 31.2829 | 29.9608 | 31.8531 | 27.6867 | 30.5032 |
| **miR-126-3p** | 27.9156 | 27.9459 | 24.5576 | 25.5747 | 26.8666 | 28.1221 | 24.0998 | 30.23599 | 26.3161 | 25.3084 | 25.8112 | 23.4901 | 23.4939 | 23.8991 | 28.3537 | 27.9195 | 25.6769 | 15.6623 | 25.0316 | 26.1422 | 26.2833 | 23.9166 | 25.3833 |
| **miR-128-3p** | 32.4353 | 29.8447 | 28.7690 | 29.6289 | 31.7348 | 31.6374 | 27.9803 | 33.49646 | 29.9508 | 29.4097 | 29.1858 | 28.3926 | 27.8360 | 28.7056 | 34.4297 | 33.3841 | 29.5035 | 14.2450 | 29.6029 | 29.8125 | 31.7214 | 25.9976 | 29.8139 |
| **miR-130b-3p** | 34.1269 | 30.8614 | 28.9331 | 31.3755 | 33.6268 | 33.8426 | 28.8314 | UND | 30.1938 | 29.4604 | 28.9647 | UND | 27.9350 | 29.7095 | 33.0242 | 35.8116 | 28.7356 | 14.3516 | 31.3628 | 31.2661 | 32.5060 | 26.6631 | 30.0217 |
| **miR-132-3p** | 33.9045 | 30.9179 | 32.5821 | 31.3500 | 33.6097 | UND | 30.1996 | UND | 30.7484 | 31.2894 | 30.8722 | 30.7228 | 30.2923 | 33.1237 | 33.4210 | 39.2683 | 30.7023 | 14.8817 | 31.9893 | 31.1950 | 32.3970 | 27.4398 | 31.9613 |
| **miR139-5p** | 32.8173 | 30.1781 | 29.1230 | 30.0940 | 31.0062 | 32.7269 | 28.7086 | 34.75026 | 29.0814 | 30.0134 | 31.6323 | 29.8155 | 28.4800 | 30.3071 | 31.7785 | 30.8852 | 30.7831 | 14.3293 | 28.9339 | 30.5587 | 29.6558 | 26.3209 | 29.9482 |
| **miR-142-5p** | 29.5759 | 26.8728 | 27.0272 | 26.8245 | 29.3928 | 29.7565 | 25.3108 | 30.84381 | 27.5871 | 25.7062 | 25.5686 | 25.2733 | 25.1439 | 24.5750 | 28.9591 | 27.1476 | 25.0831 | 14.1905 | 26.1939 | 27.6468 | 27.4021 | 24.1708 | 26.4769 |
| **miR-142-3p** | 28.6977 | 30.9835 | 28.2956 | 28.6465 | 31.7046 | 32.2855 | 25.5213 | UND | 29.3048 | 26.2736 | 28.3312 | 26.8866 | 26.5022 | 26.5338 | 33.4130 | 30.6427 | 27.7494 | 14.2380 | 29.4569 | 29.7539 | 31.3850 | 16.8688 | 29.1310 |
| **miR145-5p** | 30.5474 | 33.7904 | 29.5900 | 29.5102 | 34.4440 | 34.0401 | 28.4092 | 37.17762 | 32.7530 | 29.4974 | 31.5582 | 27.6962 | 27.7336 | 28.5585 | UND | 39.0432 | 32.5627 | 14.3639 | 32.5906 | 34.7892 | 33.4439 | 26.2781 | 31.7517 |
| **miR146a-5p** | 29.3829 | 25.5907 | 26.7604 | 27.5968 | 28.4591 | 29.4661 | 26.4884 | 33.86776 | 27.7977 | 26.8899 | 27.8616 | 26.0780 | 25.5974 | 26.1225 | 30.6030 | 31.1688 | 25.4692 | 14.1855 | 25.9264 | 27.0983 | 29.7544 | 24.6048 | 27.6266 |
| **miR-146b-5p** | 33.4998 | 28.1001 | 28.8721 | 31.1236 | 32.8460 | 31.9502 | 28.8583 | UND | 30.3776 | 30.1370 | 28.3754 | 28.4591 | 27.9551 | 28.6683 | 32.4903 | 33.4434 | 28.7951 | 14.2591 | 28.7759 | 29.8145 | 31.8713 | 26.9939 | 31.0991 |
| **miR-147a** | UND | 36.7370 | 37.1292 | UND | 37.9061 | UND | 35.4622 | UND | 36.7923 | 35.8448 | UND | UND | 35.6982 | 38.0079 | UND | UND | 33.9720 | UND | 35.6434 | UND | 34.6847 | 29.3171 | UND |
| **miR-148a-3p** | 28.2950 | 25.8795 | 25.9843 | 26.9737 | 28.5764 | 28.5757 | 25.8301 | 29.57673 | 26.8069 | 25.9170 | 25.1455 | 25.4472 | 25.6936 | 26.5995 | 27.6208 | 29.5911 | 22.6812 | 14.1810 | 26.3907 | 26.1404 | 29.7359 | 22.9245 | 27.6856 |
| **miR-150-5p** | 27.7973 | 19.3229 | 23.9235 | 23.6928 | 24.8669 | 27.1035 | 23.0206 | 26.76314 | 21.9671 | 23.9350 | 19.9110 | 22.7131 | 23.0760 | 23.3662 | 21.9573 | 25.7249 | 19.9612 | 15.6547 | 24.4952 | 25.2478 | 26.6273 | 21.9296 | 26.4105 |
| **miR-155-5p** | UND | 24.9633 | 28.2867 | 29.6466 | 29.6248 | 34.0029 | 29.5683 | 32.02152 | 28.7575 | 30.6038 | 26.4714 | 29.2933 | 30.6544 | 31.4836 | 27.3977 | 29.6428 | 24.4635 | 14.4267 | 29.4139 | 30.9071 | 30.5246 | 27.6260 | 33.1596 |
| **miR-15a-5p** | 31.4472 | 33.7188 | 27.2393 | 29.7409 | 32.6829 | 33.2638 | 26.5742 | 32.76069 | 30.3788 | 27.5720 | 26.7882 | 24.6396 | 25.6847 | 26.9629 | 32.4535 | 34.1463 | 27.2708 | 14.2281 | 28.8736 | 28.9650 | 30.4322 | UND | 28.7226 |
| **miR-15a-3p** | 33.8664 | 33.7638 | 31.5679 | 31.5577 | 32.5650 | 34.4889 | 28.8486 | 36.99352 | 32.5469 | 29.5237 | 34.1455 | 31.0064 | 30.5935 | 31.8398 | UND | 36.1100 | 34.4968 | 15.3605 | 34.2972 | 33.8720 | 30.5142 | 25.5349 | 34.4660 |
| **miR-15b-5p** | 28.7817 | 27.2222 | 24.8884 | 26.3359 | 27.7177 | 27.3468 | 24.7137 | 32.48427 | 27.4291 | 25.9576 | 23.9418 | 23.7593 | 24.3378 | 24.9541 | 27.8577 | 28.8891 | 23.7310 | 14.1694 | 25.3359 | 25.1978 | 29.3978 | 22.7568 | 25.6289 |
| **miR-16-5p** | 25.7913 | 24.2525 | 20.8159 | 23.0004 | 25.6149 | 26.4971 | 21.5937 | 31.17287 | 23.5515 | 21.7860 | 20.1618 | 18.2258 | 20.5123 | 22.1994 | 26.3666 | 28.7068 | 20.4421 | 15.6107 | 23.8286 | 24.4263 | 27.8198 | 20.8607 | 22.4898 |
| **miR-17-5p** | 28.0080 | 28.6146 | 24.6689 | 26.6871 | 29.6368 | 29.8401 | 25.3650 | 32.33209 | 27.7494 | 25.7336 | 23.8190 | 22.3691 | 23.8963 | 25.3982 | 28.3124 | 28.6118 | 23.6658 | 14.1775 | 26.8154 | 27.9427 | 29.9952 | 23.6082 | 25.9470 |
| **miR-17-3p** | 36.4132 | 31.5534 | 30.3739 | 34.2203 | UND | UND | 30.5054 | UND | 32.4013 | 30.1798 | 30.7404 | 27.7386 | 29.6414 | 31.4742 | 35.7299 | UND | 31.1627 | 14.6712 | 33.3448 | 38.5418 | 32.8162 | 28.1920 | 32.8561 |
| **miR-181a-5p** | 31.9476 | 35.6446 | 30.9482 | 31.2068 | 38.1681 | UND | 29.7536 | UND | 33.3324 | 30.2482 | 31.6684 | 29.4887 | 27.9327 | 29.5418 | 36.6427 | 36.7910 | 31.2637 | 14.6130 | 30.8846 | 33.7291 | 31.6602 | 27.5722 | 31.6871 |
| **miR-181b-5p** | 31.5696 | 31.4853 | 29.4919 | 29.8396 | 30.8177 | 32.0285 | 29.0907 | UND | 30.0095 | 29.7693 | 29.1720 | 28.8199 | 28.2275 | 29.1189 | 38.1931 | 32.9852 | 29.0042 | 14.3158 | 29.6971 | 30.1309 | 29.8597 | 25.2998 | 29.8522 |
| **miR-181c-5p** | 33.1453 | UND | 31.6456 | 31.2998 | UND | UND | 29.6924 | UND | 33.8813 | 30.7322 | 33.9167 | 29.4336 | 28.1193 | 29.5221 | 34.9801 | UND | 32.2787 | 14.5957 | 31.9732 | 34.7169 | 32.5329 | 29.3833 | 31.5134 |
| **miR-181d-5p** | 33.7586 | 32.4546 | 33.2655 | 33.1947 | UND | UND | 32.7167 | UND | 32.4617 | 33.5978 | 33.4390 | 33.5552 | 33.2880 | 32.6788 | 34.3847 | 33.9607 | 32.5569 | 14.6829 | 33.9217 | 33.4803 | 32.9133 | 27.2152 | 33.9903 |
| **miR-182-5p** | 39.5793 | 34.3590 | 31.7475 | 33.5831 | 35.3062 | 33.9265 | 31.7901 | UND | UND | UND | 30.6558 | 33.8272 | 31.4880 | 32.8901 | UND | UND | 31.3538 | 15.5454 | 34.2136 | 35.0082 | 34.0059 | 28.4226 | UND |
| **miR-184** | UND | 35.9860 | UND | UND | UND | UND | 34.7321 | UND | UND | UND | UND | 38.3296 | UND | UND | UND | UND | UND | 15.4101 | UND | 38.8437 | 36.5170 | 31.3820 | UND |
| **miR-18a-5p** | 34.3418 | 32.5724 | 27.7279 | 28.9278 | 31.4642 | 32.1488 | 28.3041 | UND | 31.4933 | 28.3539 | 26.4005 | 24.9502 | 26.8348 | 27.6242 | 32.1330 | 32.8589 | 26.6473 | 14.2541 | 29.1066 | 30.7066 | 32.6029 | 26.5942 | 28.3220 |
| **miR-191-5p** | 29.5292 | 25.7319 | 25.8285 | 26.4616 | 28.9418 | 28.5255 | 25.4384 | 30.63939 | 27.4676 | 27.2958 | 24.7382 | 24.8418 | 24.7372 | 25.5196 | 28.9427 | 29.2970 | 24.8938 | 15.6967 | 26.6196 | 26.7806 | 30.0158 | 23.8219 | 25.9277 |
| **miR-195-5p** | 26.4025 | 23.9111 | 21.0846 | 23.2199 | 25.7538 | 26.4876 | 21.9727 | 30.78589 | 23.8737 | 22.2264 | 20.3757 | 18.7771 | 20.8748 | 22.5166 | 26.1581 | 28.5127 | 20.5871 | 15.6171 | 24.1758 | 24.8012 | 27.6983 | 20.7748 | 22.5250 |
| **miR-199a-5p** | 32.5091 | 32.5767 | 30.7906 | 30.8312 | 37.1159 | UND | 29.7540 | 34.20314 | 33.5372 | 28.9619 | 33.1556 | 29.1879 | 28.1929 | 28.3190 | UND | UND | 34.2892 | 14.4014 | 30.8538 | 31.5465 | 33.5249 | 28.8706 | 31.8443 |
| **miR-19a-3p** | 26.7494 | 27.5387 | 24.3051 | 25.8024 | 28.5635 | 28.5608 | 23.9606 | 32.34514 | 26.3152 | 23.7601 | 22.7909 | 21.6121 | 23.6599 | 24.4535 | 28.1659 | 29.1261 | 22.8875 | 14.1832 | 25.6772 | 26.1101 | 28.7775 | 22.5018 | 24.9508 |
| **miR-19b-3p** | 27.0355 | 27.5249 | 24.6545 | 25.8614 | 27.5161 | 27.8173 | 24.1422 | 28.30588 | 26.3201 | 24.1355 | 23.2971 | 21.8851 | 23.7723 | 24.5506 | 27.5310 | 27.7170 | 23.2891 | 14.1782 | 25.8321 | 26.3529 | 27.5159 | 16.2741 | 25.4234 |
| **miR-204-5p** | 35.1912 | 35.6279 | 31.7585 | 33.4774 | 34.3992 | 36.4938 | 32.7815 | 35.77196 | 34.1435 | 34.3604 | 33.9107 | 34.4637 | 32.6536 | 36.3266 | 36.6441 | 34.9591 | 36.3777 | 14.7924 | 34.2736 | 35.1372 | 33.5440 | 27.5074 | 34.6269 |
| **miR-20a-5p** | 27.2842 | 27.1519 | 23.3298 | 25.4115 | 28.0130 | 28.7773 | 23.9236 | 30.61106 | 26.3997 | 24.1355 | 21.9930 | 20.6588 | 22.6201 | 23.8342 | 26.5619 | 27.2271 | 21.9943 | 14.1758 | 25.7321 | 26.3759 | 28.4116 | 22.6292 | 24.7497 |
| **miR-20b-5p** | 28.2446 | 27.8307 | 24.4379 | 26.7900 | 29.6138 | 29.4239 | 25.3427 | 31.93799 | 27.4120 | 25.6346 | 23.0711 | 21.9398 | 23.9178 | 25.2998 | 27.6930 | 28.3474 | 23.0300 | 15.6855 | 26.7625 | 27.7186 | 29.5057 | 22.6982 | 25.7957 |
| **miR-21-5p** | 26.7716 | 21.5026 | 23.3896 | 23.7010 | 24.2766 | 25.6724 | 23.6099 | 26.35101 | 22.7999 | 22.9604 | 20.9482 | 22.4089 | 23.5879 | 22.7767 | 23.1354 | 25.8079 | 20.4902 | 15.6026 | 21.5131 | 22.4622 | 23.5932 | 19.6553 | 23.9192 |
| **miR-210-3p** | 34.6964 | 34.4122 | 30.4396 | 32.3290 | 34.7186 | 35.7078 | 29.5259 | 34.96127 | 32.8147 | 29.6401 | 33.7724 | 27.9041 | 29.9639 | 31.8298 | 35.3680 | 34.5163 | 31.7556 | 18.2443 | 32.5585 | 33.7094 | 31.5003 | 24.6373 | 32.6508 |
| **miR-214-3p** | 34.5206 | UND | 31.5917 | 32.2639 | 38.4776 | 39.1115 | 30.8030 | 34.41985 | 33.4748 | 32.3166 | UND | 31.9056 | 31.5745 | 35.4237 | 36.7649 | UND | 35.5375 | 15.2843 | 32.8333 | 34.8671 | 31.7348 | 27.6869 | UND |
| **miR-221-3p** | 29.8696 | 27.0130 | 26.2813 | 27.4336 | 29.4531 | 31.0028 | 25.3718 | 32.27831 | 27.6036 | 25.6036 | 28.2198 | 24.5587 | 24.7172 | 25.6191 | 32.9069 | 31.3286 | 27.6295 | 14.1819 | 26.3676 | 27.4285 | 28.7948 | 23.7511 | 27.6667 |
| **miR222-3p** | 30.2113 | 26.9254 | 26.9627 | 27.9407 | 29.3259 | 31.1861 | 26.4248 | UND | 27.6599 | 27.1472 | 27.8655 | 25.5632 | 26.5770 | 26.7124 | 32.3589 | 31.7180 | 27.0282 | 14.1941 | 27.2310 | 27.7390 | 28.9565 | 23.7985 | 27.7930 |
| **miR223-3p** | 24.1235 | 22.1268 | 21.5448 | 21.5386 | 24.5173 | UND | 20.2238 | 30.52741 | 23.1498 | 21.6746 | 23.2698 | 21.9970 | 20.9998 | 21.6406 | 25.9658 | 27.4797 | 23.3074 | 15.6448 | 22.4849 | 20.9454 | 27.8663 | 17.5938 | 23.3144 |
| **miR-23a-3p** | 26.9589 | 23.5026 | 23.7601 | 24.4947 | 26.2552 | 26.5649 | 23.1824 | 31.92827 | 25.3579 | 24.2529 | 24.7528 | 23.6907 | 22.9806 | 23.5278 | 26.4904 | 27.9313 | 23.7935 | 14.1670 | 23.9126 | 24.1332 | 25.9590 | 21.2374 | 24.8086 |
| **miR23b-3p** | 28.7279 | 25.8922 | 27.3198 | 28.6944 | 29.1938 | 30.6547 | 26.9887 | 32.43036 | 28.2938 | 27.8549 | 27.6770 | 26.6707 | 26.4297 | 26.7207 | 29.4527 | 30.8726 | 25.9432 | 14.1878 | 26.9120 | 28.6222 | 28.2312 | 25.6857 | 27.2287 |
| **miR-24-3p** | 27.3514 | 26.6826 | 25.3321 | 26.3061 | 28.3276 | 28.5019 | 24.5034 | 33.52676 | 26.8708 | 25.5027 | 27.1582 | 24.6156 | 24.5436 | 25.2024 | 30.3281 | 29.5062 | 25.5311 | 15.7140 | 25.7560 | 26.0268 | 29.3678 | UND | 26.3834 |
| **miR-25-3p** | 26.9490 | 24.6540 | 24.2064 | 25.3565 | 26.3943 | 27.5929 | 24.6553 | 31.84111 | 25.5241 | 25.1818 | 22.8897 | 21.7559 | 23.8789 | 25.5830 | 26.9439 | 28.9006 | 23.4091 | 15.6431 | 25.2303 | 25.5722 | 28.6714 | 21.7655 | 24.5078 |
| **miR-26a-5p** | 27.5187 | 23.9078 | 24.5473 | 25.3506 | 26.9887 | 27.6253 | 24.3355 | 29.56398 | 25.7910 | 25.8383 | 22.5326 | 23.5003 | 23.5708 | 23.7061 | 24.9096 | 25.9276 | 22.0856 | 15.6587 | 24.2035 | 25.6068 | 27.4503 | 23.3697 | 24.6303 |
| **miR-26b-5p** | 27.7184 | 23.5646 | 24.4028 | 25.6674 | 26.9183 | 27.4440 | 24.5257 | 29.54582 | 26.1610 | 26.2498 | 21.8438 | 22.8432 | 23.6024 | 23.9066 | 24.5824 | 26.4535 | 21.7385 | 14.1855 | 24.3793 | 25.3330 | 27.8592 | 22.0198 | 24.7219 |
| **miR-27a-3p** | 26.6268 | 25.7869 | 24.9106 | 25.8317 | 28.2845 | 27.7730 | 23.8209 | 31.89091 | 26.6057 | 24.1033 | 25.6938 | 24.3982 | 24.5067 | 24.4747 | 28.2437 | 28.5486 | 24.0863 | 14.1795 | 25.2232 | 25.1156 | 27.5088 | 21.5793 | 26.4888 |
| **miR-27b-3p** | 30.7996 | 27.4291 | 26.9941 | 28.7129 | 30.0194 | 30.1511 | 27.1111 | 33.42757 | 28.9198 | 27.4346 | 27.9942 | 26.9614 | 26.6706 | 26.9411 | 30.3949 | 31.2401 | 26.3704 | 14.2064 | 27.1826 | 28.1083 | 31.3633 | 24.0241 | 28.3825 |
| **miR-28b-3p** | 34.7296 | 30.7367 | 32.9262 | 34.9086 | 36.9698 | 33.9357 | 29.4186 | UND | 30.9788 | 29.9857 | 28.6793 | 28.7298 | 28.4761 | 29.1764 | 31.1936 | 32.2380 | 28.2308 | 14.3796 | 31.2891 | 33.6269 | UND | 28.8412 | 31.6678 |
| **miR-29a-3p** | 28.9343 | 23.9559 | 24.4977 | 25.6039 | 28.1155 | 28.7755 | 23.7367 | 28.44373 | 24.7584 | 23.1725 | 22.7496 | 22.5172 | 23.7519 | 23.7099 | 25.0128 | 28.3101 | 21.7410 | 14.1717 | 24.1341 | 26.1365 | 27.4513 | 19.5501 | 27.7048 |
| **miR-29b-3p** | 31.3633 | 25.5559 | 26.8078 | 27.6339 | 29.6905 | 30.6059 | 26.4789 | 30.55324 | 26.9955 | 26.1556 | 23.5098 | 24.9875 | 26.2931 | 26.2286 | 25.9486 | 29.1831 | 23.1811 | 15.6576 | 26.4672 | 28.1919 | 30.5476 | 23.9406 | 29.1135 |
| **miR-29c-3p** | 28.3187 | 23.7606 | 23.9044 | 25.3261 | 27.4448 | 28.9524 | 23.3448 | 28.39873 | 24.7101 | 22.8936 | 21.7954 | 22.0688 | 23.4197 | 23.1878 | 24.7748 | 27.2769 | 21.3979 | 15.6281 | 23.8588 | 25.5413 | 27.1362 | 20.3736 | 26.8999 |
| **miR-30a-5p** | 28.6463 | 26.4195 | 25.3757 | 26.4937 | 28.6114 | 29.5351 | 24.7919 | 34.67260 | 26.7051 | 25.4932 | 26.8872 | 23.1079 | 24.3400 | 25.1628 | 31.4614 | 29.3367 | 27.3222 | 14.1779 | 26.4829 | 27.3259 | 29.3181 | 23.0170 | 26.4322 |
| **miR-30b-5p** | 30.4298 | 30.9201 | 28.4459 | 28.6267 | 31.1656 | 32.0064 | 27.5207 | 33.44831 | 30.1847 | 29.5005 | 28.1745 | 27.5691 | 27.1811 | 27.0746 | 31.5028 | 29.8062 | 28.3124 | 14.2276 | 28.7490 | 29.9284 | 30.5972 | 26.2994 | 28.4926 |
| **miR-30c-5p** | 28.8333 | 28.9041 | 28.1132 | 28.2945 | 29.7034 | 30.8469 | 26.8161 | 32.49979 | 29.0102 | 28.1446 | 28.1719 | 26.8960 | 26.3791 | 26.2189 | 30.4201 | 29.0904 | 27.9472 | 15.7714 | 27.5789 | 28.6764 | 29.3374 | 24.7666 | 27.3886 |
| **miR-30d-5p** | 28.6528 | 26.8614 | 26.1594 | 26.8804 | 28.9791 | 30.2875 | 26.0131 | 33.48731 | 27.9432 | 26.8869 | 27.5011 | 24.6794 | 25.4597 | 26.1511 | 33.9259 | 29.9177 | 27.9058 | 14.1866 | 26.9248 | 27.8820 | 30.5335 | 24.6852 | 26.9350 |
| **miR-30e-5p** | 27.5173 | 26.8652 | 25.3223 | 26.4935 | 28.3993 | 29.8486 | 24.4430 | 32.40950 | 26.6968 | 25.1068 | 26.8733 | 22.6978 | 24.1608 | 24.9682 | 31.3526 | 29.3960 | 26.9175 | 14.1724 | 26.5621 | 26.9704 | 29.0261 | 23.0167 | 26.4397 |
| **miR-31-5p** | 38.0788 | 36.4306 | 33.0281 | 35.8916 | 34.0969 | 38.1252 | 31.7062 | UND | 38.1647 | 33.5796 | 37.2875 | 32.9280 | 33.6847 | 39.2627 | 35.3308 | 37.6705 | 38.8180 | 15.7302 | 33.7470 | 36.2419 | 33.4197 | 27.7820 | 38.4000 |
| **miR-326** | 26.6195 | 26.4009 | 26.3801 | 29.7201 | 31.6986 | 33.4356 | 26.9943 | UND | 30.5916 | 27.5852 | 34.1937 | 27.2009 | 28.3116 | 28.9339 | 39.8597 | 35.0148 | 33.2197 | 14.7142 | 30.5613 | 32.4067 | 29.9386 | 24.7871 | 26.6687 |
| **miR-331-3p** | 34.3643 | 37.9407 | 31.3783 | 31.8553 | 37.1905 | 37.8750 | 29.5515 | UND | 33.2987 | 30.5221 | 33.8443 | 28.2828 | 29.4936 | 29.7575 | 33.6220 | 33.2024 | 31.8642 | 14.6838 | 32.8420 | 32.2570 | 32.8946 | 28.7797 | 32.3055 |
| **miR-335-5p** | 35.6361 | 30.1500 | 30.5598 | 32.0698 | 33.1678 | 36.6888 | 30.9976 | 33.20850 | 33.8065 | 31.8567 | 31.6887 | 30.9101 | 30.2994 | 30.6447 | 33.3794 | 32.8609 | 31.3272 | 14.6243 | 31.1142 | 32.1988 | 33.4335 | 27.3477 | 31.7978 |
| **miR-342-3p** | 30.2932 | 25.8359 | 27.5977 | 27.7403 | 28.5174 | 30.6039 | 26.6636 | 30.68615 | 26.7288 | 26.8837 | 25.3992 | 27.1747 | 26.7925 | 27.5515 | 27.6414 | 28.5089 | 25.3524 | 14.2084 | 27.9786 | 28.6489 | 29.8481 | 23.1651 | 28.2532 |
| **miR-346** | 32.6029 | 31.3312 | 28.0306 | 29.1088 | 29.9769 | 32.3107 | 26.4756 | 39.37021 | 30.4253 | 26.9846 | 34.2016 | 27.9994 | 28.0250 | 30.2885 | 33.6102 | 33.7839 | 31.9367 | 14.4014 | 29.5978 | 31.9382 | 28.5851 | 24.5786 | 31.5702 |
| **miR-34a-5p** | 34.8341 | 32.9561 | 29.5265 | 31.4703 | 34.4240 | 34.3158 | 29.1279 | 33.51807 | 30.5412 | 29.2212 | 32.9911 | 28.8706 | 29.5177 | 28.7958 | 32.9097 | UND | 29.5754 | 14.2221 | 29.7382 | 28.4423 | 28.9993 | 22.9427 | 34.2657 |
| **miR-365a-3p** | 31.3317 | 31.6081 | 29.4901 | 28.9894 | 30.9744 | 31.4648 | 28.7337 | UND | 30.1835 | 30.4608 | 30.6420 | 29.9681 | 28.9738 | 28.8167 | UND | 34.1733 | 30.4205 | 14.2997 | 30.9287 | 29.2280 | 33.6771 | 25.9281 | 30.4463 |
| **miR-423-5p** | 28.5534 | 25.6460 | 26.8306 | 27.6199 | 27.7628 | 29.7446 | 26.8726 | 28.71244 | 26.7037 | 27.8956 | 25.7680 | 25.5848 | 26.7126 | 27.1500 | 26.3108 | 28.3137 | 24.9858 | 15.7359 | 26.4685 | 27.3812 | 29.3876 | 24.3264 | 26.9707 |
| **miR-574-3p** | 29.6934 | 28.9774 | 27.3948 | 28.0897 | 28.6464 | 30.5103 | 26.4529 | 33.88734 | 28.5011 | 27.5951 | 30.8452 | 28.4919 | 26.8555 | 29.5231 | 33.0167 | 32.6738 | 29.1889 | 14.2370 | 28.1607 | 30.5669 | 27.3511 | 23.4310 | 30.3785 |
| **miR-92a-3p** | 25.4840 | 24.4772 | 22.8792 | 23.9909 | 25.3409 | 26.8892 | 23.9638 | 31.49058 | 24.4749 | 24.1870 | 22.8517 | 20.9110 | 22.9162 | 24.2376 | 26.8875 | 27.5621 | 23.4055 | 15.6659 | 24.4410 | 25.2584 | 25.9812 | 12.6103 | 22.9628 |
| **miR-93-5p** | 28.4373 | 28.1436 | 25.3806 | 27.0221 | 29.5098 | 30.3587 | 25.4302 | UND | 27.4850 | 25.9006 | 24.6667 | 22.6054 | 24.5442 | 25.9251 | 30.7269 | 30.8906 | 24.7629 | 15.7231 | 27.7982 | 27.7522 | 31.4208 | 23.7025 | 26.3857 |
| **miR-98-5p** | 33.8636 | 31.8099 | 32.2985 | 31.7569 | 32.6061 | UND | 30.1133 | 33.01495 | 31.9383 | 32.2007 | 30.8511 | 31.0940 | 29.6478 | 29.4454 | 32.8072 | 33.2535 | 29.7125 | 14.3628 | 29.4308 | 31.9764 | 39.7050 | 28.6839 | 30.4417 |
| **miR-99a-5p** | 31.4234 | 39.7339 | 29.7131 | 30.4068 | 32.1856 | UND | 29.9154 | UND | 32.6696 | 31.2161 | 30.7566 | 29.3153 | 28.9387 | 30.4833 | 34.0737 | 34.9644 | 32.2011 | 14.4340 | 33.2903 | 31.9843 | 32.1897 | 27.6908 | 31.6358 |
| **SNORD95*** | 30.7815 | 25.3448 | 27.0030 | 26.5506 | 29.3518 | 31.8466 | 27.3020 | 31.37589 | 26.1180 | 25.9318 | 24.8590 | 26.7779 | 26.7755 | 27.6845 | 28.8302 | 31.9221 | 22.9736 | 14.3322 | 31.6065 | 34.7562 | 30.1776 | 16.7245 | 31.0264 |
| **SNORD96a*** | 32.5331 | 25.4271 | 27.9822 | 27.9085 | 29.6952 | 33.2160 | 28.9010 | 34.25436 | 27.5451 | 27.7743 | 26.7232 | 27.2826 | 29.4933 | 29.5939 | 30.1742 | 31.5136 | 25.5519 | 14.5432 | 30.7418 | 32.1182 | 30.6986 | 26.7876 | 32.4127 |
| **RNU6-2*** | 34.0270 | 33.4687 | 32.1719 | 35.2495 | UND | 37.4062 | 30.2748 | UND | 32.9803 | 31.5182 | 33.3377 | 30.3481 | 31.6968 | 32.4963 | 35.5356 | 32.9366 | 32.2733 | 15.3206 | 32.8914 | 31.7663 | 36.9195 | 27.7150 | 34.8490 |

Ct values obtained in qT-PCR reaction utilizing T-Cell & B-Cell Activation miScript miRNA PCR Array (QIAGEN). 1-22 - CLL patients, Cal - calibrator (pooled serum collected from 8 healthy volunteers), * - selected sno/snRNA controles used for data normalization using the ΔΔC_T_ method, UND - undetermined.
