# Supplementary material for: Expression of circulating miRNAs associated with lymphocyte differentiation and activation in CLL—another piece in the puzzle
Source: Ann Hematol. 2016 Oct 12;96(1):33–50. doi: 10.1007/s00277-016-2840-6 (PMC5203831; doi:10.1007/s00277-016-2840-6)
Supplement: Supplementary file 3 — Functional gene grouping – according to manufacturer of T-Cell & B-Cell Activation miScript miRNA PCR Array (QIAGEN) (DOCX 13 kb) [file 277_2016_2840_MOESM3_ESM.docx]

**Expression of circulating miRNAs associated with lymphocyte differentiation and activation in CLL – another piece in the puzzle.**

Annals of Hematology.

Agata A. Filip^1^, Anna Grenda^1^, Sylwia Popek^1^, Dorota Koczkodaj^1^, Małgorzata Michalak-Wojnowska^1^, Michał Budzyński^1^, Ewa Wąsik-Szczepanek^2^, Szymon Zmorzyński^1^, Agnieszka Karczmarczyk^3^, Krzysztof Giannopoulos^3^.

1. Department of Cancer Genetics, Medical University of Lublin, Poland

2. Department of Hematooncology and Bone Marrow Transplantation, Medical University of Lublin, Poland

3. Department of Experimental Hematooncology, Medical University of Lublin, Poland

Correspondence to: Agata A. Filip, Department of Cancer Genetics, Medical University of Lublin, Radziwiłłowska 11, 20-080 Lublin, Poland. Tel/fax: +48 81 4486100, e-mail: aafilip@hotmail.com

**Table S3.**

**Functional Gene Grouping** – according to manufacturer of T-Cell & B-Cell Activation miScript miRNA PCR Array (QIAGEN)

**T-Cell Differentiation:**

***Double Negative (CD4-/CD8-):*** let-7d-5p, let-7e-5p, miR-126-3p, miR-128, miR-146b-5p, miR-15a-5p, miR-17-5p, miR-17-3p, miR-181c-5p, miR-191-5p, miR-19a-3p, miR-199a-5p, miR-20a-5p, miR-20b-5p, miR-221-3p, miR-222-3p, miR-223-3p, miR-28-5p, miR-29c-3p, miR-30e-5p, miR-342-3p, miR-423-5p, miR-93-5p, miR-98-5p.

***Double Positive (CD4+/CD8+):*** let-7b-5p, miR-181a-5p, miR-181b-5p, miR-181d, miR-19b-3p.

**CD4+ Naive:** miR-132-3p, miR-146a-5p, miR-182-5p, miR-184, miR-25-3p, miR-326, miR-92a-3p.

***CD8+ Naive:*** let-7a-5p, let-7c, let-7f-5p, let-7g-5p, miR-130b-3p, miR-139-5p, miR-142-3p, miR-150-5p, miR-155-5p, miR-15a-3p, miR-16-5p, miR-26a-5p, miR-26b-5p, miR-29b-3p, miR-30b-5p, miR-30c-5p, miR-30d-5p.

***CD8+ Effector:*** miR-147a, miR-148a-3p, miR-18a-5p, miR-27a-3p, miR-27b-3p.

***CD8+ Memory:*** let-7i-5p, miR-106b-5p, miR-142-5p, miR-15b-5p, miR-17-5p, miR-21-5p, miR-23a-3p, miR-23b-3p, miR-24-3p, miR-29a-3p, miR-31-5p.

**B-Cell Differentiation:**

***Naive:*** let-7a-5p, let-7b-5p, let-7d-5p, let-7g-5p, let-7i-5p, miR-101-3p, miR-132-3p, miR-142-3p, miR-142-5p, miR-150-5p, miR-181c-5p, miR-195-5p, miR-204-5p, miR-214-3p, miR-221-3p, miR-222-3p, miR-223-3p, miR-29b-3p, miR-30e-5p, miR-331-3p, miR-92a-3p.

***Germinal Center:*** miR-106b-5p, miR-130b-3p, miR-132-3p, miR-148a-3p, miR-15a-5p, miR-15b-5p, miR-16-5p, miR-17-5p, miR-17-3p, miR-181a-5p, miR-181b-5p, miR-191-5p, miR-19a-3p, miR-19b-3p, miR-210, miR-23b-3p, miR-25-3p, miR-28-5p, miR-30d-5p, miR-93-5p, miR-98-5p.

***Memory:*** miR-100-5p, miR-125b-5p, miR-145-5p, miR-146a-5p, miR-155-5p, miR-21-5p, miR-23a-3p, miR-24-3p, miR-26a-5p, miR-26b-5p, miR-27a-3p, miR-27b-3p, miR-29a-3p, miR-29c-3p, miR-30b-5p, miR-30c-5p, miR-34a-5p.

**Differentially Expressed in Tregs:** miR-100-5p, miR-125b-5p, miR-146a-5p, miR-181c-5p,miR-20b-5p, miR-21-5p, miR-31-5p, miR-335-5p, miR-365a-3p, miR-99a-5p.

**T-Cell Activation:** let-7e-5p, let-7g-5p, let-7i-5p, miR-106b-5p, miR-139-5p, miR-142-5p, miR-146a-5p, miR-146b-5p, miR-155-5p, miR-15a-5p, miR-15a-3p, miR-181a-5p, miR-181c-5p,miR-195-5p, miR-20b-5p, miR-214-3p, miR-223-3p, miR-23a-3p, miR-23b-3p, miR-25-3p,miR-26a-5p, miR-26b-5p, miR-27a-3p, miR-27b-3p, miR-29a-3p, miR-29b-3p, miR-29c-3p,miR-30a-5p, miR-30b-5p, miR-30e-5p, miR-342-3p, miR-346, miR-574-3p, miR-98-5p.
